# Prevention of acute kidney injury by low intensity pulsed ultrasound via anti-inflammation and anti-apoptosis

**DOI:** 10.1038/s41598-020-71330-1

**Published:** 2020-08-31

**Authors:** Chih-Kang Chiang, Jui-Zhi Loh, Ting-Hua Yang, Kuo-Tong Huang, Cheng-Tien Wu, Siao-Syun Guan, Shing-Hwa Liu, Kuan-Yu Hung

**Affiliations:** 1 Institute of Toxicology, College of Medicine, National Taiwan University, Taipei, Taiwan; 2 Departments of Integrated Diagnostics and Therapeutics and Internal Medicine, College of Medicine and Hospital, National Taiwan University, Taipei, Taiwan; 3 Department of Otolaryngology, College of Medicine, National Taiwan University, Taipei, Taiwan; 4 Department of Internal Medicine, College of Medicine and Hospital, National Taiwan University, Taipei, Taiwan; 5 Institute of Nutrition, College of Biopharmaceutical and Food Sciences, China Medical University, Taichung, Taiwan; 6 Master Program of Food and Drug Safety, China Medical University, Taichung, Taiwan; 7 Institute of Nuclear Energy Research, Atomic Energy Council, Taoyuan, Taiwan; 8 Department of Medical Research, China Medical University Hospital, China Medical University, Taichung, Taiwan; 9 Department of Pediatrics, National Taiwan University Hospital, Taipei, Taiwan

**Keywords:** Diseases, Health care, Nephrology

## Abstract

The therapeutic effects of low intensity pulsed ultrasound (LIPUS) on renal ischemia/reperfusion injury (IRI) with acute kidney injury (AKI) are still unclear. A renal tubule cell model under H_2_O_2_ or hypoxia/reoxygenation (H/R) conditions with or without LIPUS pre-treatment (1 MHz, 30 and 100 mW/cm^2^, 15 min) was used to test the in vitro effects of LIPUS. An AKI mouse model of unilateral IRI with nephrectomy of the contralateral kidney for 48 h with or without LIPUS treatment (3 MHz, 100 mW/cm^2^, 20 min/day) 5 day before IRI were used to investigate the in vivo effects of LIPUS. LIPUS significantly protected the renal tubule cell viability and prevented inflammatory signals against H_2_O_2_ challenge. LIPUS could inhibit the apoptosis-related molecular signals and increase the protein levels of endogenous antioxidant enzymes, α-Klotho, and Sirt1 in renal tubule cells after H/R challenge. LIPUS alleviated the increases in the serum levels of blood urea nitrogen, creatinine, and cystatin C, renal pathological changes and apoptosis-related molecular signals, and impaired antioxidant enzymes in AKI mice. The IRI-induced inflammatory responses in the kidneys and spleens could be reversed by LIPUS. These findings suggest that LIPUS treatment displays the benefits for renal protection in IRI-induced AKI mice.

## Introduction

Therapeutic ultrasound has been developed for clinical application and obtained acceptance. Ultrasound with high and low intensities can transmit and induce physiological changes in target tissues via thermal and non-thermal influences, respectively^1^. Low-intensity pulsed ultrasound (LIPUS) is a kind of ultrasound with non-thermal effect and outputs in a pulse wave mode, which is delivered at a much lower intensity (< 3 W/cm^2^) than traditional ultrasound energy^2^. LIPUS in the diagnostic intensity range has been approved by the US FDA for the accelerated healing of fresh fractures and for the treatment of established non-union^3^. Studies have demonstrated that LIPUS can promote bone regeneration and enhance osteogenesis and improve the cartilage repair^4–6^. LIPUS is recognized as a non-invasive and safe therapeutic tool for bone fracture treatment. LIPUS has recently been demonstrated to effectively prevent the cerebral ischemia/reperfusion injury in a mouse model^7^. Ogata et al. recently showed the efficacy of LIPUS on cardiac dysfunction in a mouse model by enhancing myocardial angiogenesis and attenuating perivascular fibrosis^8^. Gigliotti et al. found that a pulsed ultrasound with a mechanical index of 1.2 treatment possessed therapeutic potential for the prevention of acute kidney injury (AKI) via stimulating a splenic cholinergic anti-inflammatory pathway^9^. The microbubble contrast agents have further been suggested to improve the sensitivity and specificity of ultrasound for diagnosing or possibly treating kidney disease^10^.

Ischemia/reperfusion injury (IRI) is a common cause of AKI, characterized by restriction of renal blood supply followed by blood flow restoration and re-oxygenation, which occurs upon conditions such as infarction, organ transplantation, or major surgery^11–13^. The recovery process in AKI patients is often incomplete. However, the preventive or therapeutic effect of LIPUS on renal IRI still remains to be investigated. There is still lack of suitable preventive or therapeutic intervention for AKI. We therefore hypothesized that LIPUS possesses the potential for AKI prevention. We tried to investigate the protective effects of LIPUS on IRI-induced AKI in vitro and in vivo. A renal tubule cell model under H_2_O_2_ or hypoxia/reoxygenation (H/R) conditions with or without LIPUS pre-treatment was used to test the in vitro effects of LIPUS. An AKI mouse model of unilateral IRI with nephrectomy of the contralateral kidney for 48 h with or without LIPUS treatment were used to investigate the in vivo effects of LIPUS on IRI-related AKI.

## Results

### LIPUS pretreatment alleviated acute renal cell injury in the renal tubule cell models

**Figure 1:**
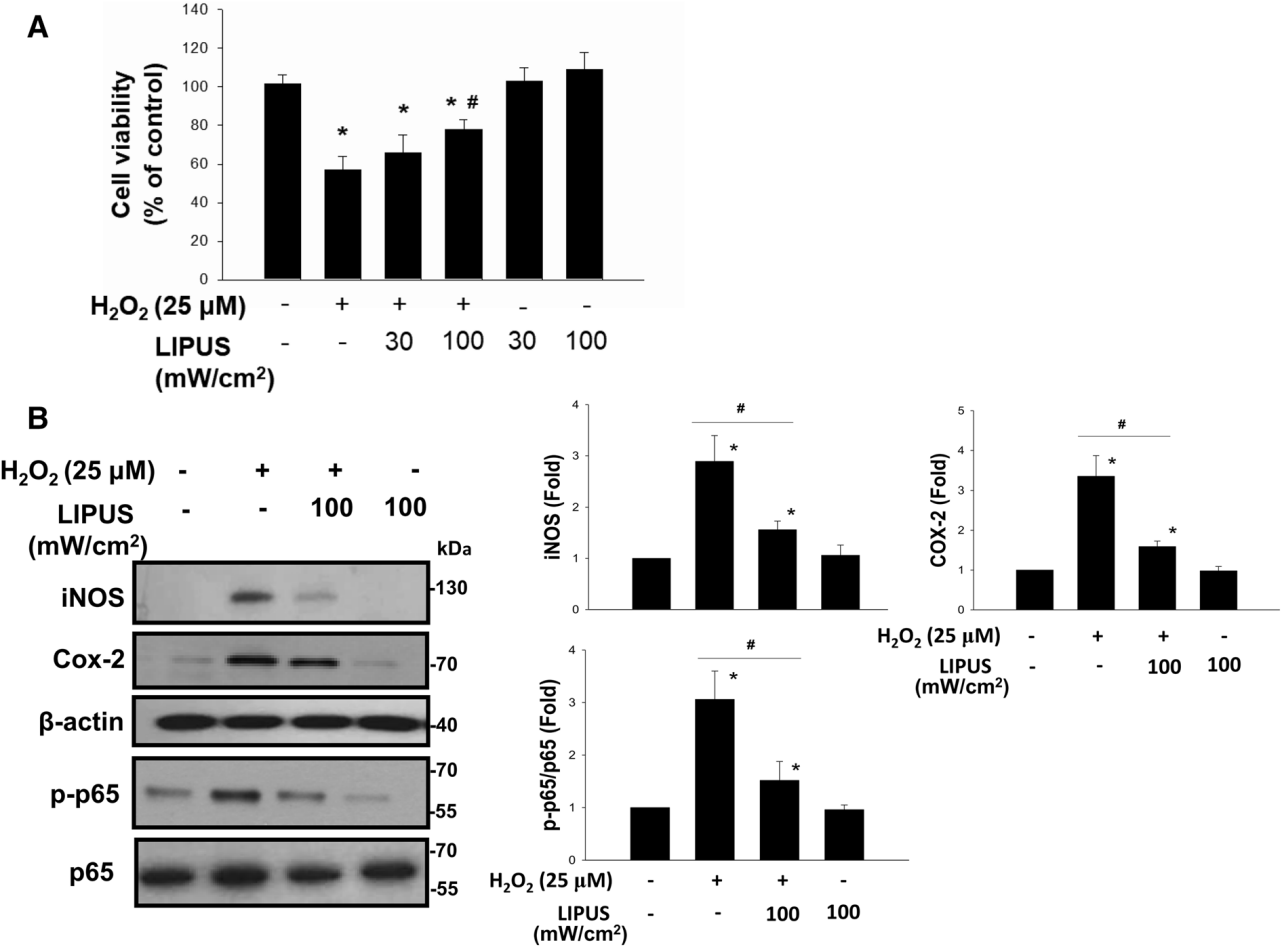
Effects of LIPUS on H_2_O_2_-induced cell viability inhibition and inflammatory signals in renal tubule NRK52E cells. Cells were treated with H_2_O_2_ (25 μM) for 24 h with or without LIPUS (30 or 100 mW/cm^2^) treatment. LIPUS was performed to the cell culture for a period of 15 min before the beginning of the experiment (H_2_O_2_). (**A**) The cell viability was determined by MTT assay. Both 30 and 100 mW/cm^2^ LIPUS 30 were performed. (**B**) The 100 mW/cm^2^ LIPUS was performed. The protein levels of inducible NO synthase (iNOS), cyclooxygenase-2 (Cox-2), phosphorylated NFκB-p65 (p-p65), and p65 were determined by Western blotting. Data are presented as means ± SEM for three to five independent experiments. *p < 0.05 versus control. #*p* < 0.05 versus H_2_O_2_ alone.

To clarify the effects of LIPUS on oxidative stress-related renal cell injury and inflammatory signals, an in vitro H_2_O_2_-induced renal cellular injury model to mimic oxidative stress-mediated cell injury was used^14^. As shown in Fig. 1A, after treatment with H_2_O_2_, the cell viability was significantly decreased, which could be significantly reversed by LIPUS at 100 mW/cm^2^ intensity (39.3% reduction), but not at 30 mW/cm^2^ intensity (8.1% reduction). The protein levels of iNOS, COX-2, and phosphorylated NFκB-p65 in H_2_O_2_-treated NRK-52E cells were also markedly increased, which could be significantly reversed by LIPUS (100 mW/cm^2^) pre-treatment (Fig. 1B; 46.4% reduction in iNOS, 53.8% reduction in COX-2, 50.1% reduction in p-p65/p-65). LIPUS alone did not affect the NRK-52E cell viabilty (Fig. 1A).

**Figure 2:**
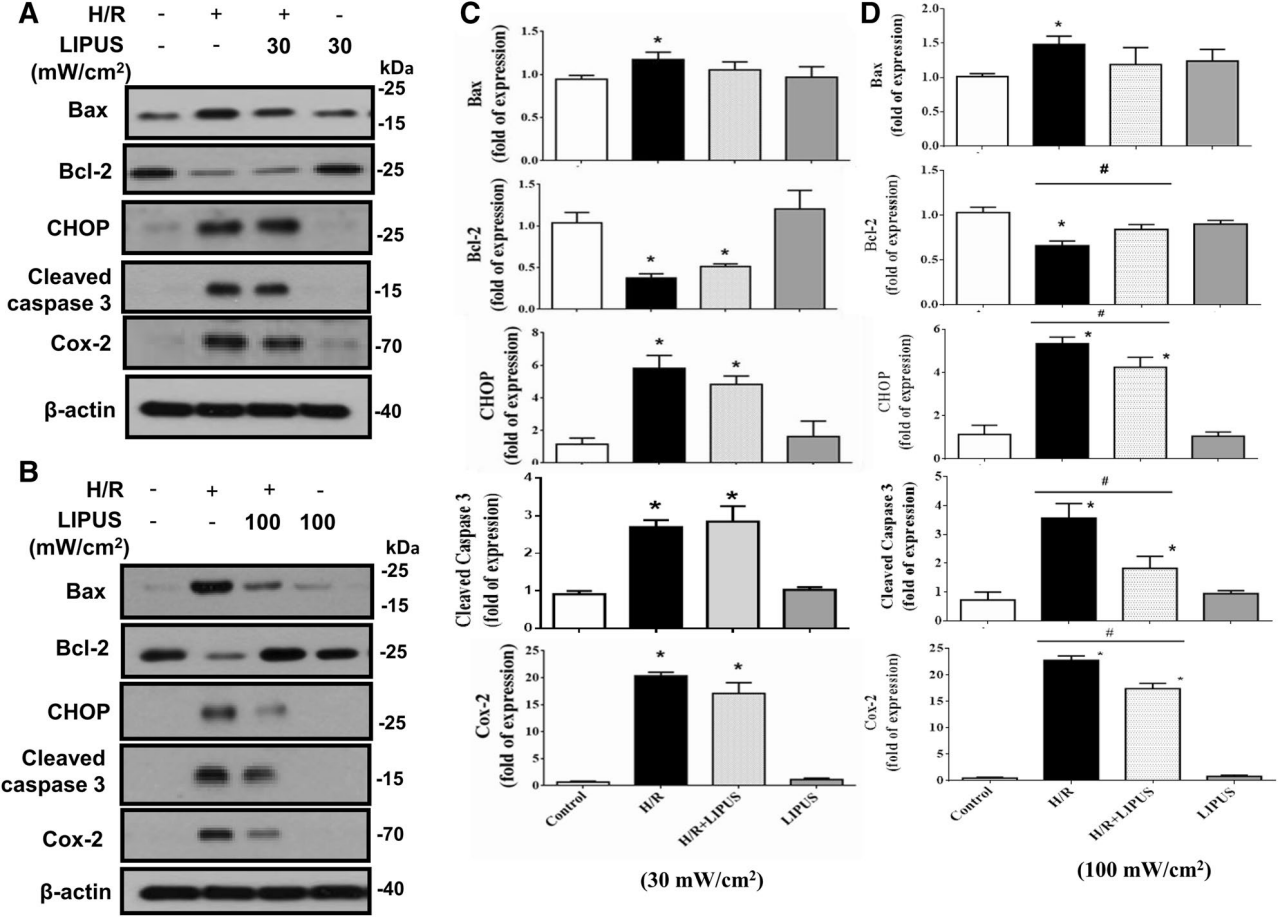
The effects of LIPUS on apoptosis-related molecular signals in renal tubular NRK52E cells after hypoxia/reoxygenation (H/R) treatment. Cells were treated with or without LIPUS [30 (**A**, **C**) and 100 (**B**, **D**) mW/cm^2^] treatment. LIPUS was performed to the cell culture for a period of 15 min before the beginning of the experiment (H/R). The protein levels of Bax, Bcl-2, CHOP, cleaved caspase-3, and COX-2 in NRK-52E cells after H/R (6 h hypoxia/24 h reoxygenation) were determined by Western blotting. The data for quantification were shown in C and D. Data are presented as means ± SEM for three to five independent experiments. *p < 0.05 versus control. #*p* < 0.05 versus H/R alone.

**Figure 3:**
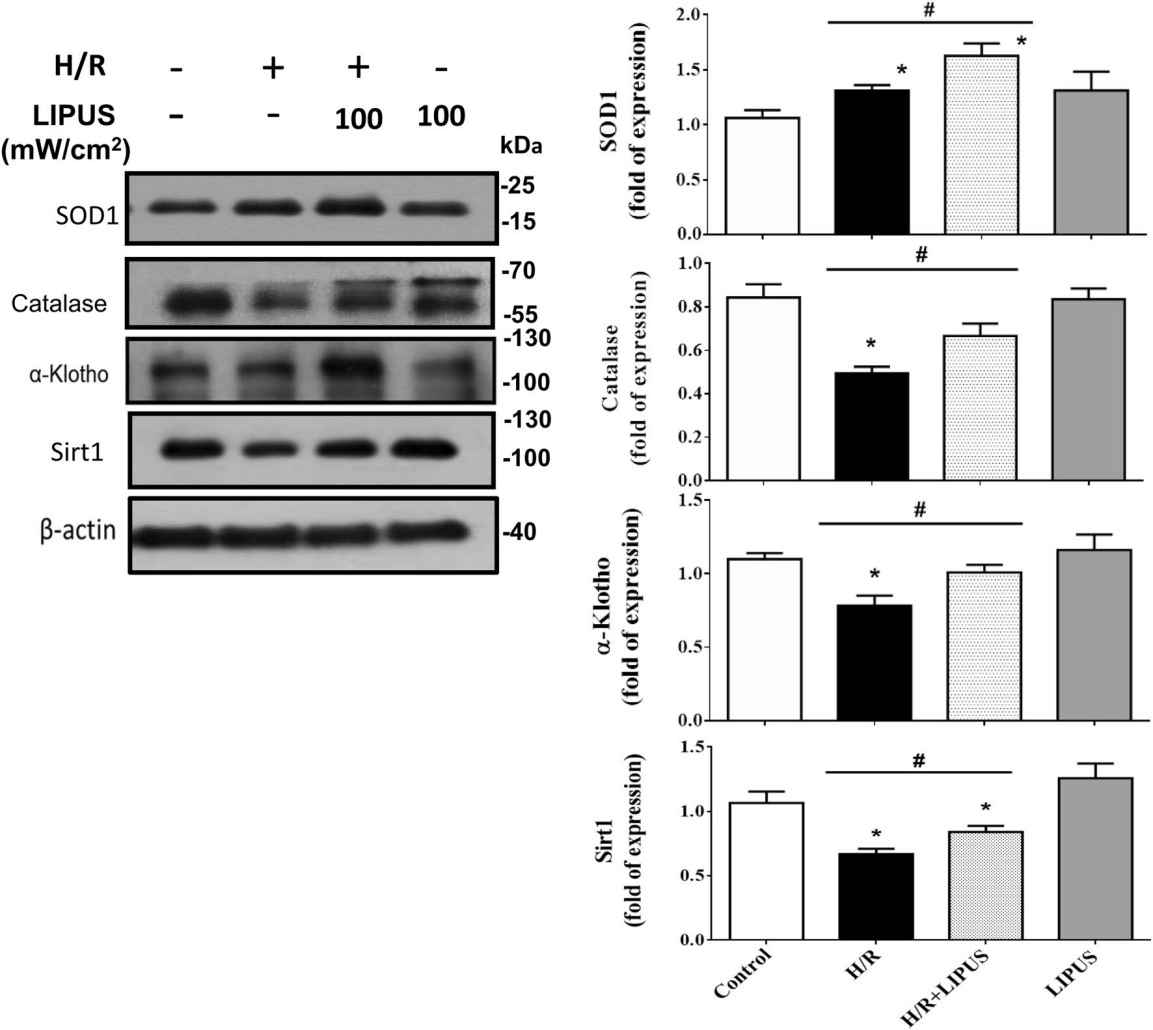
The effects of LIPUS on endogenous antioxidant enzymes and α-Klotho and Sirt1 in renal tubular NRK52E cells after hypoxia/reoxygenation (H/R) treatment. Cells were treated with or without LIPUS (100 mW/cm^2^) treatment. LIPUS was performed to the cell culture for a period of 15 min before the beginning of the experiment (H/R). The protein levels of antioxidant enzymes SOD1 and catalase and α-Klotho and Sirt1 in NRK-52E cells after H/R (6 h hypoxia/24 h reoxygenation) were determined by Western blotting. Data are presented as means ± SEM for three to five independent experiments. *p < 0.05 versus control. #*p* < 0.05 versus H/R alone.

It has been found that the H_2_O_2_ production can be induced in NRK-52E cells after hypoxia/reoxygenation (H/R)^15^. We further used a hypoxia/reoxygenation (H/R) model to test the protective effects of LIPUS on renal tubule cell injury. As shown in Fig. 2, after H/R, the protein levels of Bax, CHOP, cleaved caspase-3, and COX-2 were markedly increased and the Bcl-2 protein level was decreased in NRK-52E cells, which could be significantly reversed by pre-treatment with LIPUS at 100 mW/cm^2^ intensity (Fig. 2B,D; 30.1% reduction in Bax, 49.2% increase in Bcl-2, 30.9% reduction in CHOP, 51.4% reduction in cleaved caspase-3, 28.9% reduction in COX-2), but not at 30 mW/cm^2^ intensity (Fig. 2A,C). Moreover, LIPUS at 100 mW/cm^2^ intensity pre-treatment significantly increased the protein levels of SOD1, catalase, α-Klotho, and Sirt1 in NRK-52E cells after H/R (Fig. 3; 36.0% increase in SOD1, 29.2% increase in catalase, 26.7% increase in α-Klotho, 25.0% increase in Sirt1). Unexpectedly, H/R alone significantly increased SOD1 protein level and LIPUS treatment increased it further (Fig. 3).

After treatment with H/R, the cell viability was significantly decreased, which could be significantly reversed by LIPUS at 100 mW/cm^2^ intensity (Control, 99.20 ± 2.02%; LIPUS, 102.02 ± 1.49%; H/R, 70.59 ± 2.59%; H/R + LIPUS, 88.20 ± 2.28%, n = 7, *p* < 0.05 H/R vs H/R + LIPUS; Supplementary Fig. 1).

### LIPUS treatment alleviated IRI-associated AKI in a mouse model

**Figure 4:**
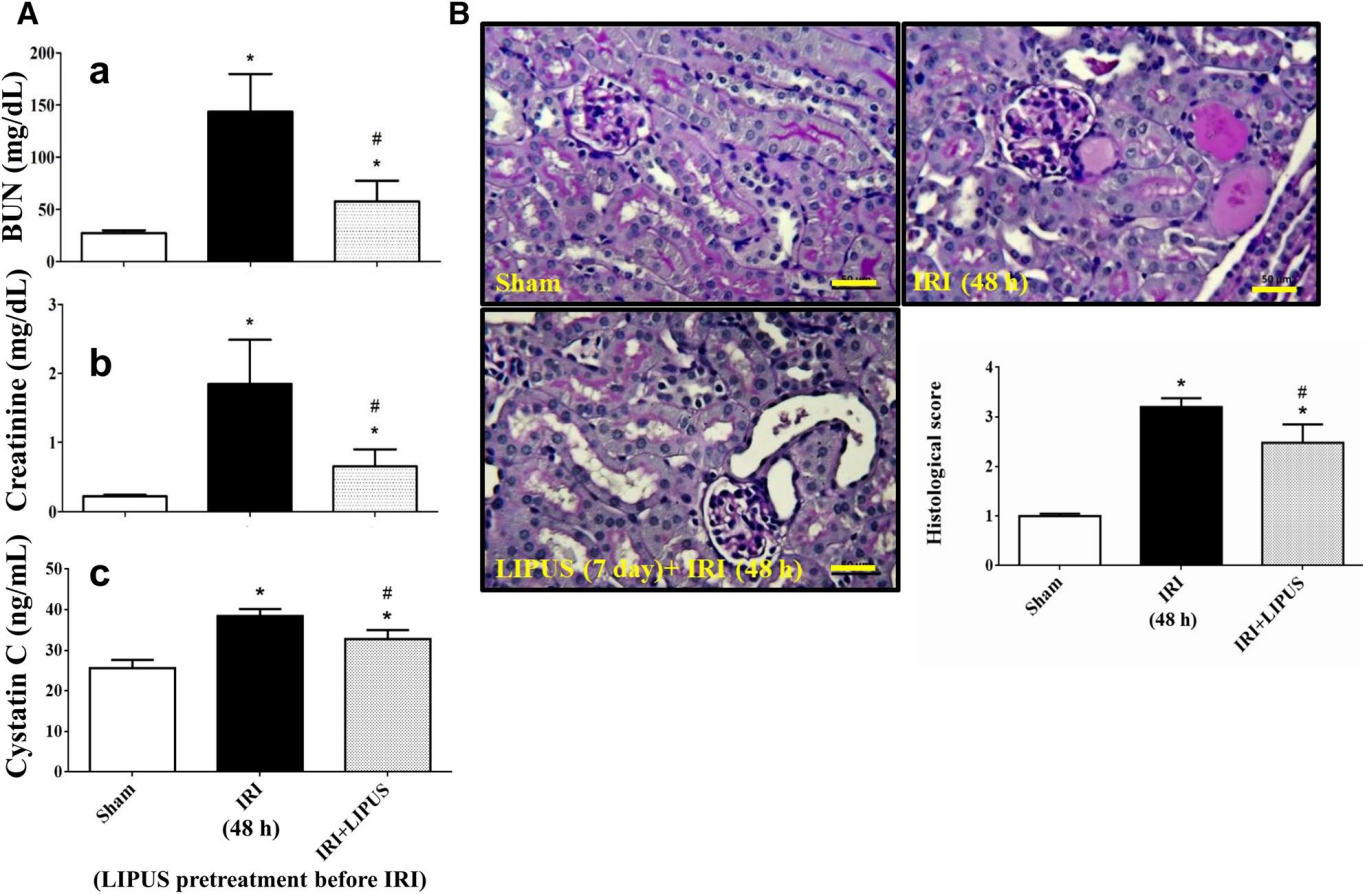
Effects of LIPUS on the renal injury in an acute kidney injury (AKI) mouse model of unilateral ischemia/reperfusion injury (IRI) with contralateral nephrectomy. Animals were euthanized 48 h after IRI. The LIPUS treatment was performed before IRI procedure and after IRI until the day of euthanization. (**A**) The serum blood urea nitrogen (BUN, a), creatinine (b), and cystatin C (CYS C, c) levels were shown. (**B**) Renal tissues were stained with Periodic Acid-Schiff (PAS) and pathological changes were observed under light microscope. Histological score was also recorded. Data are presented as mean ± SEM (n = 8). *p < 0.05 versus sham; # *p* < 0.05 versus IRI alone. Scale bar: 50 μm.

An AKI mouse model of unilateral IRI with contralateral nephrectomy in the presence or absence of LIPUS treatment was induced. The LIPUS treatment consisted of 20 min/day for 5 days prior to IRI and continued daily until mice were euthanized. LIPUS (30 or 100 mW/cm^2^) alone did not alter the parameters measured 48 h or 7 days after treatment in sham control mice (Supplementary Figs. 2 and 3). The levels of serum creatinine, BUN, and cystatin C were markedly increased in mice with IRI-associated AKI (Fig. 4A). LIPUS treatment could significantly reverse these increased biochemical parameters (Fig. 4A; 60.7% reduction in BUN, 58.3% reduction in creatinine, 26.3% reduction in cystatin C). The histopathological changes in renal tissues were observed by using PAS staining and the histological scores were evaluated. As shown in Fig. 4B, IRI mice exhibited severe renal injury, including tubular cell death, and tubule dilation, which could be partially but significantly reversed by LIPUS (100 mW/cm^2^) treatment (31.3% reduction).

**Figure 5:**
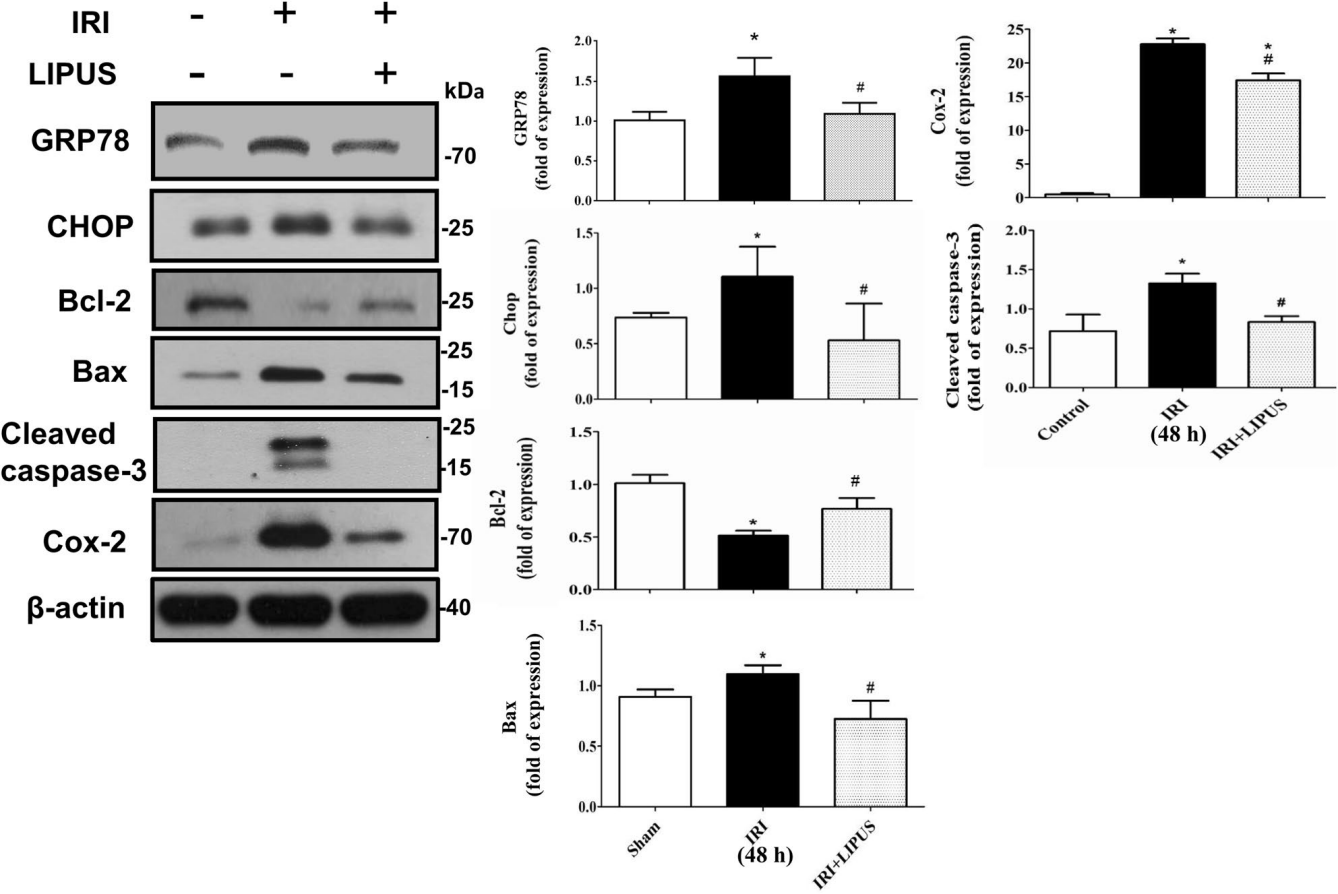
Effects of LIPUS on the renal apoptosis-related molecular signals in an AKI mouse model. Animals were euthanized 48 h after IRI. The LIPUS treatment was performed before IRI procedure and after IRI until the day of euthanization. The protein levels of GRP78, Chop, Bcl-2, Bax, Cox-2, and cleaved caspase-3 in the kidneys were determined by Western blot, which were quantified by densitometry and normalized by β-actin levels. Data are presented as mean ± SEM (n = 8). *p < 0.05 versus sham; #*p* < 0.05 versus IRI alone.

We further tested the levels of apoptosis-related signaling molecules in the kidneys. As shown in Fig. 5, the protein levels of GRP78, Chop, Bax, and cleaved caspase-3 were increased and Bcl-2 protein level was decreased in the kidneys of IRI mice, which could be significantly reversed by LIPUS treatment (26.7% reduction in GRP78, 53.6% reduction in CHOP, 50.1% increase in Bcl-2, 34.8% reduction in Bax, 42.3% reduction in cleaved caspase-3). Moreover, the increased Cox-2 protein level in the kidney of IRI mice could also be significantly reversed by LIPUS treatment (Fig. 5; 26.8% reduction).

**Figure 6:**
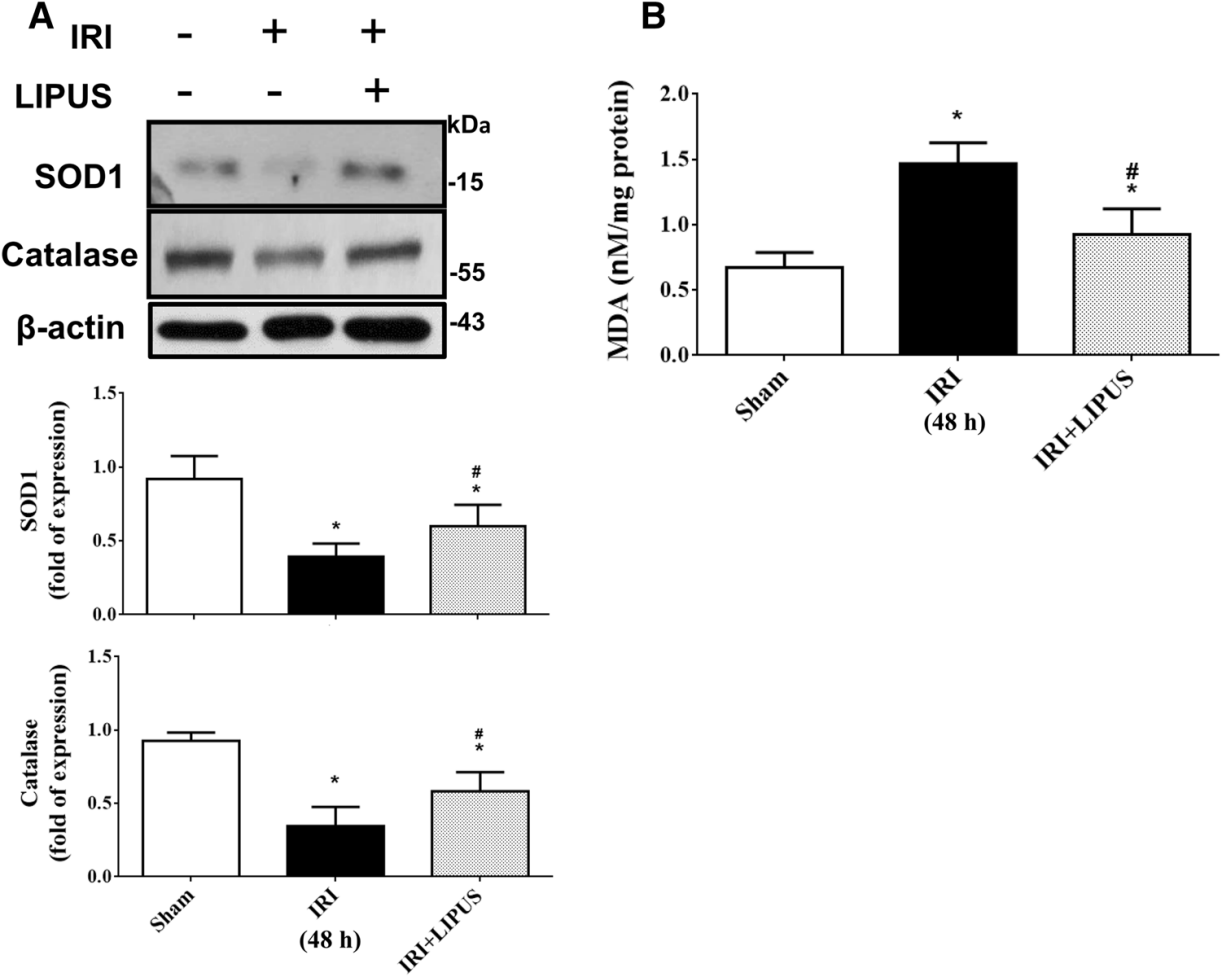
Effects of LIPUS on the renal endogenous antioxidant enzymes and lipid peroxidation in an AKI mouse model. The LIPUS treatment was performed before IRI procedure and after IRI until the day of euthanization. The antioxidant enzymes SOD1 and catalase protein levels (**A**) and malondialdehyde (MDA) levels (**B**) in the kidneys were shown. The protein level was determined by Western blot and quantified by densitometry, which was normalized by β-actin. Data are presented as mean ± SEM (n = 8). *p < 0.05, versus Sham; #*p* < 0.05, versus IRI alone.

Endogenous antioxidants including catalase and superoxide dismutase (SOD) are important in scavenging reactive oxygen species (ROS). The decreased level of antioxidant enzymes in renal tissues was observed after IRI^16^. We also found that the protein levels of SOD1 and catalase were decreased (Fig. 6A) and the MDA levels (Fig. 6B) were increased in the kidneys of AKI mouse model, which could be significantly reversed by LIPUS treatment (Fig. 6; 47.1% increase in SOD1, 45.7% increase in catalase, 39.2% reduction in MDA).

**Figure 7:**
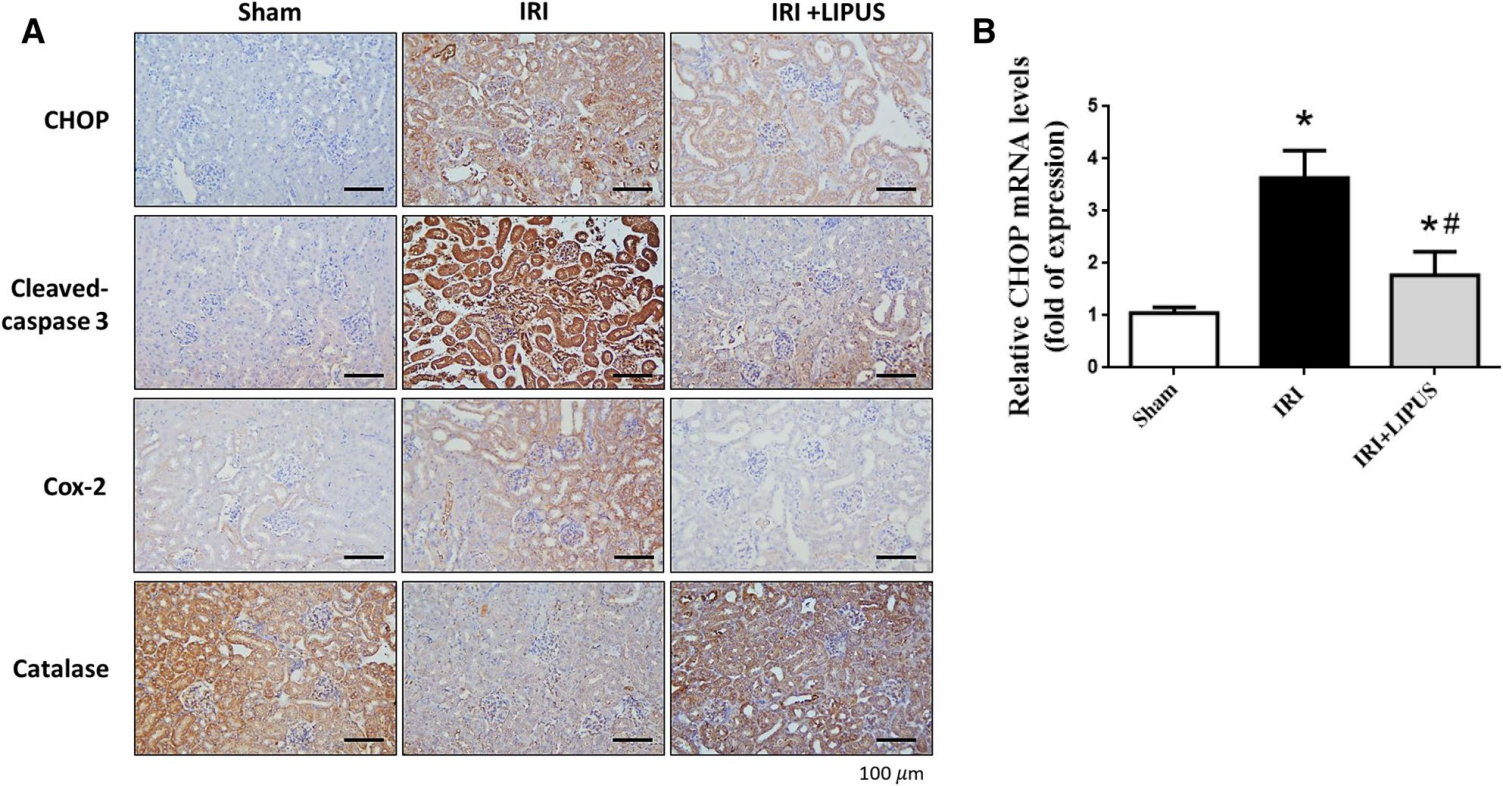
Effects of LIPUS on the some signaling molecules in the kidneys of an AKI mouse model. The LIPUS treatment was performed before IRI procedure and after IRI until the day of euthanization. The changes in immunohistochemistry and real-time PCR were determined. (**A**) The stainings for CHOP, cleaved caspase-3, Cox-2, and catalase in the kidneys were shown. The representative images of at least three independent experiments were shown. Scale bar = 100 μm. (**B**) The mRNA expression for CHOP in the kidneys was shown. Two reference genes of β-actin and Gapdh were used. Data are presented as mean ± SEM (n = 4–6). *p < 0.05, versus Sham; #*p* < 0.05, versus IRI alone.

To confirm the effects of LIPUS on the signaling molecules in the kidneys of IRI-induced AKI mice, the immunohistochemistry and real-time PCR were utilized to determine the effects of LIPUS on mRNA and protein expression/localization. As shown in Fig. 7A, the stains for CHOP, cleaved caspase-3, and Cox-2 were increased and the staining for catalase was decreased in the kidneys of IRI mice, which could be effectively reversed by LIPUS treatment. Moreover, the mRNA expression of CHOP was markedly increased in the kidneys of IRI mice, which could also be significantly reversed by LIPUS pretreatment (Fig. 7B; 52.8% reduction in CHOP mRNA).

**Figure 8:**
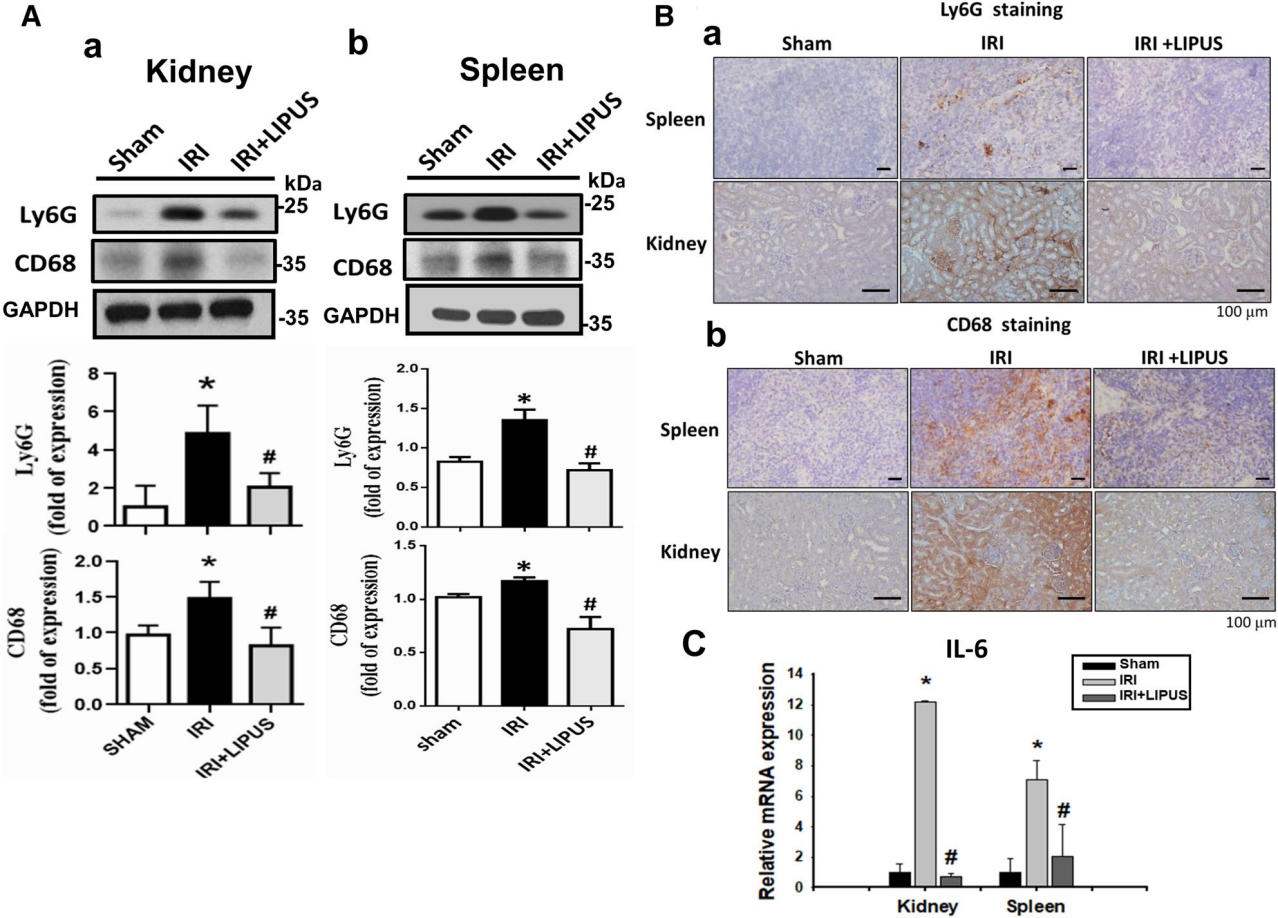
Effects of LIPUS on the inflammatory cell infiltration in the kidneys and spleens of an AKI mouse model. The LIPUS treatment was performed before IRI procedure and after IRI until the day of euthanization. The Ly6G (a neutrophil marker) and CD68 (a macrophage marker) protein levels in the kidneys (**A**-a) and spleens (A-b) were shown. The protein level was determined by Western blot and quantified by densitometry, which was normalized by GAPDH. The stainings of Ly6G (**B**-a) and CD68 (B-b) in the kidneys and spleens were determined by immunohistochemistry. The representative images of at least three independent experiments were shown. Scale bar = 100 μm. The IL-6 mRNA expression in the kidneys and spleens was determined by real-time PCR (**C**). Two reference genes of β-actin and Gapdh were used. Data are presented as mean ± SEM (n = 4–6). *p < 0.05, versus Sham; #*p* < 0.05, versus IRI alone.

We further tested the effects of LIPUS on the inflammatory cell infiltration in the kidneys and spleens of IRI-induced AKI mice. As shown in Fig. 8, the protein levels of Ly6G (a neutrophil marker) and CD68 (a macrophage marker) were significantly increased in the kidneys (Fig. 8A-a) and spleens (Fig. 8A-b) of IRI mice, which could be significantly reversed by LIPUS treatment (kidney: 59.6% reduction in Ly6G, 46.7% reduction in CD68; spleen: 51.8% reduction in Ly6G, 40.9% reduction in CD68). LIPUS treatment could also reverse the increased Ly6G staining by immunohistochemistry in the kidneys and spleens of IRI mice (Fig. 8B). Moreover, the mRNA expression of IL-6 was significantly increased in the kidneys and spleens of IRI mice, which could be significantly reversed by LIPUS treatment (Fig. 8C; kidney: 86.7% reduction; spleen: 71.4% reduction).

## Discussion

LIPUS treatment is low-cost and non-invasive^17^. LIPUS is known to be a form with medium frequency ultrasound (0.7–3 MHz) and delivers at a lower intensity (< 3 W/cm^2^) than traditional ultrasound energy^2^. Gigliotti et al. have shown that IRI-induced AKI in an animal model can be prevented by a pulsed ultrasound using the parameters as follows: a mechanical index of 1.2 and duration of 1 s were applied once every 6 s for 2 min^9^. The LIPUS conditions in the present study are 1 MHz frequency and 30 and 100 mW/cm^2^ intensity (15 min) for cell model and 3 MHz frequency and 100 mW/cm^2^ intensity (20 min daily) for animal model with pulse repetition rate of 100 Hz and pulse duration of 5 ms that the mechanical index for LIPUS (1 MHz frequency) applied by Sonicator-740 has been estimated to be 0.3^18^. In addition, our preliminary study showed that LIPUS (30 or 100 mW/cm^2^) alone did not alter the parameters measured 48 h or 7 days after treatment in sham control mice (Supplementary Figs. 2 and 3). Moreover, the mechanical index for LIPUS used in this study is very low. Therefore, there is a safe condition for LIPUS exposure in this study. Following the 3Rs principle for animal experiments, we did not further investigate the effects of LIPUS alone on sham control mice.

The accumulation of excessive ROS causes the cellular oxidative stress, mitochondrial dysfunction, and initiation of cell death^19^. Renal ischemia degraded cellular ATP to hypoxanthine, which is converted to xanthine by xanthine oxidase and generates superoxide radical in the presence of molecular oxygen^20,21^. Superoxide can be converted to hydrogen peroxide by superoxide dismutase (SOD) and finally is converted to water and molecular oxygen by catalase. Oxidative stress has been suggested to be a key mediator of chronic kidney disease (CKD) and nephrotoxic and ischemic AKI; the ROS generation may lead to intra-renal inflammation results^22^. ROS is capable of upregulating the NF-κB activation and the protein levels of Cox-2, iNOS, MCP-1, and IL-1β^22,23^. The H_2_O_2_ generation and catalase activity have been shown to be increased and decreased, respectively, in NRK-52E cells during H/R challenge^15^. In the present study, we found that LIPUS could increase the protein levels of SOD1 and catalase and reverse the H_2_O_2_-reduced cell viability and H_2_O_2_-increased NF-κB activation and protein levels of iNOS and Cox-2 in NRK-52E cells during H/R challenge. Moreover, the restoration of catalase and SOD1 protein levels and lipid peroxidation (increased MDA level) in the kidneys of IRI-associated AKI model by LIPUS treatment was also observed. These results suggest that retrieval of cell antioxidant defense system can protect renal cells from further ROS damage during H/R or IRI-associated AKI challenge.

For cell viability under conditions of H_2_O_2_ and H/R, we found that the protective effect of LIPUS was more effective in the H/R model than in the H_2_O_2_ model. Perhaps there was too much injury in the latter. Moreover, we unexpectedly found that H/R alone significantly increased SOD1 protein level and LIPUS treatment increased it further (Fig. 3). Chronic hypoxic preconditioning has been suggested to upregulate the SOD activity and contents that SOD may play a role in protecting hypoxia adapted rats from oxidative stress^24^. However, in our IRI mouse model, the protein level of SOD1 in the kidney was significantly decreased. A further investigation for this issue may be needed.

It has been suggested that ER and mitochondria cooperate to participate in cell death^25^. The Bcl-2/Bax ratio is known to be a rheostat to regulate anti-oxidant pathway and apoptotic state^26^. An oxidative stress induced by IRI has been found to be involved in the renal proximal tubular cell apoptosis via the activation of both mitochondrial stress and ER stress pathways, which could be inhibited by berberine, an antioxidant^27^. CHOP is a main regulator of ER stress-induced apoptosis^28^. CHOP knockout cells and animals have been shown to be markedly protected from cell death and renal injury caused by ER stress, implicating down-regulation of CHOP is advantageous in an IRI-associated AKI mouse model^15^. The persistent ER stress and protein misfolding-initiated ROS cascades have been suggested to play the pathological roles in the multiple human disorders^29^. CHOP deficiency has been found to decrease the IRI-induced apoptosis and lipid peroxidation and increase the activity of endogenous antioxidants in the kidneys^15^. In the present study, we found that LIPUS treatment effectively alleviated the upregulation of CHOP, Bax, and cleaved caspase-3 protein levels and the downregulation of Bcl-2 protein level in NRK-52E cells during H/R challenge and in the kidneys of IRI-AKI mice, indicating LIPUS treatment protects renal cells from IRI via inhibiting CHOP signaling and increasing Bcl-2/Bax ratio pathways.

The cellular mechanisms for LIPUS stimulation are still unclear. The therapeutic effects of LIPUS on tissues have been theorized by transmitting pressure waves to produce micromechanical strains in biological tissues that may result in biochemical events and accelerate tissue healing^17^. Bandow et al. have shown that LIPUS treatment triggers chemokines and receptor activator of NF-κB ligand (RANKL) expressions in osteoblasts through the angiotensin II type 1 receptor as a mechanoreceptor^30^. However, the really cellular mechanism(s) for preventive effects of LIPUS treatment on IRI-associated AKI still needs a further investigation.

The previous studies have shown that several volatile anesthetics possess the anti-inflammatory effect to protect against renal IRI as compared to pentobarbital or ketamine anesthesia^31,32^. It may be a concern about using isoflurane as it is reported to protect against renal IRI. In the present study, mice were anesthetized with isoflurane mixed with oxygen during the LIPUS treatment. However, mice were anesthetized with ketamine and xylazine during renal IRI surgery. Moreover, mice from all groups had the same experimental procedure for anesthesia. Therefore, the isoflurane anesthesia may not affect the positive results of LIPUS on AKI mice.

Gigliotti and Okusa have mentioned that the spleen tissue is a forgotten organ in AKI; they have suggested that the spleen tissue plays an important role in sepsis-associated AKI and IRI-induced AKI^33^. Gigliotti et al. have also shown that IRI-induced AKI can be prevented via the activation of a splenic anti-inflammatory pathway by a pulsed ultrasound^9^. They have further found that the injured kidneys can be protected from IRI in mice transferred by the splenocytes isolated from ultrasound-treated mice^34^. In the present study, we found that the increases in the protein levels of Ly6G and CD68 and the mRNA expression of IL-6 in the kidneys and spleens of IRI-induced AKI could be significantly reversed by LIPUS pretreatment. According to the studies of Gigliotti and colleagues, the preventive effect of LIPUS on IRI-induced renal injury may be attributed to a splenic anti-inflammatory mechanism.

The studies of Gigliotti et al. showed that both kidneys were exposed to pulsed ultrasound with a mechanical index of 1.2 in a similar manner in a bilateral renal IRI-induced AKI mouse model; they compared the insonating right kidney versus left kidney and found a robust protection only when the left kidney was insonated, leading to a splenic mechanism^9,34^. In the present study, we used an unilateral IRI with nephrectomy of the contralateral kidney AKI mouse model that the right kidney was nephrectomized and only the left abdominal region of IRI mouse was insonated by LIPUS with a low mechanical index. However, the ultrasound imaging was not employed with this LIPUS application that kidney insonation and not that of other abdominal/retroperitoneal organs is assumed. Based on these limitations, our findings implies that insonating the mouse left abdominal region causes a reduction in AKI, and this protective effect may be not as robust as that observed in other studies.

In conclusions, LIPUS treatment may improve the outcome of renal IRI-associated AKI that there are associative changes with respect to treatment. LIPUS treatment may be potentially applied to an alternative non-invasive therapeutic intervention on renal injury therapy or serve as an auxiliary tool for management of AKI. The evaluation of longer follow-up times after AKI or chronic kidney disease (CKD) for LIPUS efficacy is an important issue and deserves investigation in the future.

## Methods

### Cell culture

A normal rat renal proximal tubule cell line NRK-52E was obtained from the Bioresource Collection and Research Center (Hsinchu, Taiwan). Cells were cultured in DMEM supplemented with 5% fetal bovine serum and antibiotics (100 U/mL penicillin and 0.1 mg/mL streptomycin) at 37 °C in 5% CO_2_. In some experiments, the cell culture in a hypoxia/reoxygenation (H/R) condition was performed. The oxygen content was reduced to 0.2% to provide hypoxic condition using an Anaerocult A mini system (Merck, Whitehouse Station, NJ). After 6 h of hypoxia, cells were removed from the hypoxic condition followed by a medium change for 24 h reoxygenation.

### Cell viability assay

Cells were treated with H_2_O_2_ (25 μM) for 24 h or H/R (6 h/24 h) with or without LIPUS (100 mW/cm^2^) pre-treatment (15 min). Culture medium was added with 0.5 mg/mL 3-(4,5-dimethylthiazol-2-yl)-2,5-diphenyltetrazolium bromide (MTT; Sigma-Aldrich, St. Louis, MO, USA) for 2 h incubation, and then 100 μL dimethyl sulfoxide (DMSO) was added to dissolve blue formazan crystals. The plates were incubated at room temperature for 30 min, and then an absorbance at 570 nm was detected by a spectrophotometer.

### Animals and experimental protocol

Adult C57BL/6J male mice (6-week-old) were obtained from the Laboratory Animal Center of the college of Medicine, National Taiwan University. Mice were kept at room temperature with light/dark cycle and were given access to standard rodent chow and tap water ad libitum. The animal study was conducted with the approval of the Animal Research Committee of College of Medicine, National Taiwan University and complied with the guideline for the care and use of laboratory animals. Mice were treated humanely and with regard for alleviation of suffering. Animals were randomized into the sham, IRI, and IRI + LIPUS groups, and were anesthetized with Ketamine (100 mg/kg, i.p.) and Xylazine (10 mg/kg, i.p.) before surgery. The AKI model was performed by unilateral IRI with nephrectomy as previously described by Skrypnyk et al. whom observed IRI with 90–100% survival in mice^35^. In the IRI group with or without LIPUS treatment, the left renal artery was isolated and clamped for 30 min using a non-traumatic artery clamp, followed by reperfusion. During surgery and recovery from anesthesia for both IRI and sham procedure, the body temperature of mice was maintained at 37 ± 0.5 °C (rectal temperature) with a heat plate and heat lamp. The sham control mice underwent all steps of the surgery except for clamping the artery. The nephrectomy for right kidney was performed 24 h before the mice were euthanized. In the IRI + LIPUS group, mice were treated with LIPUS (20 min/day) for 5 days before IRI procedure and after IRI until the day of euthanization. A schematic representation of time course for the induction of IRI mice with or without LIPUS treatment was shown in Supplementary Fig. 4. The mice were euthanized 48 h after IRI. The levels of serum creatinine, blood urea nitrogen (BUN), and cystatin C and the histological examination were evaluated.

### LIPUS treatment

LIPUS was applied using Sonicator-740, a clinically available and portable therapeutic ultrasound device from Mettler Electronics (Anaheim, CA, USA). In an animal model, a 3 MHz single-element focused transducer with 1 cm^2^ surface area was used to generate the LIPUS. Mice were anesthetized with isoflurane mixed with oxygen during the LIPUS procedure. Mice were kept warm during LIPUS with a heat plate and heat lamp. The transducer was placed at the left lateral region of mouse abdomen and the signal was transmitted through coupling gel. The mouse received 20 min/day of LIPUS exposure at a spatial-peak temporal-average intensity of 100 mW/cm^2^. The pulse settings had pulse repetition rate of 100 Hz with pulse width of 5 ms and 5 ms between pulses (pulse space = 10 ms). The duty cycle was 50% and the on time to off time ratio was 1:1 (5 ms on and 5 ms off), which were chosen to obtain minimal thermal effects^2,36^. In a renal cell model, LIPUS generated from a 5 cm^2^ transducer with 1 MHz frequency and the 30 or 100 mW/cm^2^ intensity was performed to the cell culture for a period of 15 min before the beginning of the experiment (H_2_O_2_ or H/R). LIPUS was transmitted from the plane transducer to the bottom of the cell culture plate in which ultrasound transmission gel was used to cover the area between the transducer and the plate to maximize the transmission of the ultrasound. The LIPUS parameters were set according to the previous literatures and our preliminary experiments. The mechanical index for LIPUS (1 MHz frequency) applied by Sonicator-740 has been estimated to be 0.3^18^. Mice from all groups had the same experimental procedure for anesthesia. The groups of sham control and IRI alone were exposed to all aspects of the LIPUS procedure (including fur removal) except for the actual insonation.

### Serum biochemical measurement

Serum creatinine and blood urea nitrogen (BUN) levels in mice were determined by a commercially available clinical chemistry analyzer (Roche, Rotkreuz, Switzerland). Cystatin C levels were measured using a mouse ELISA kit (Immunology Consultants Laboratory, Portland, OR, USA).

### Histological analysis

The whole kidney tissues were fixed in 10% formalin and embedded in paraffin. To determine histological changes, the 4-μm-thick tissue sections were stained with Periodic acid-Schiff (PAS). The histological analysis was performed by a pathologist of the Laboratory Animal Center of the college of Medicine, National Taiwan University, with a double blind fashion. Fifteen randomly selected fields per section were observed. Tubular injuries, including renal tubule dilation, tubular epithelial injury, and cast formation, were graded with a score from 0 to 4 (0, no change; 1, change affecting less than 25% of the field; 2, change affecting 25–50% of the field; 3, change affecting 50–75% of the field; 4, change affecting more than 75% of the field)^37^.

### Immunoblotting

The protein level in the renal tubule cells and kidney tissues was determined by Western blotting as previously described^15^. The sodium dodecyl sulfate (SDS)-PAGE was used to separate equal amount of proteins, and then transferred electrophoretically onto polyvinylidene difluoride (PVDF) membranes. The membranes were incubated with primary antibodies for GRP78 (#3183), Chop (#2895), Bax (#2772), Bcl-2 (#15071), cleaved caspase-3 (#9664), Sirtuin-1 (Sirt1; #8469), phosphorylated NFκB-p65 (#3033) (Cell Signalling, Danvers, MA, USA), catalase (#ab16731), superoxide dismutase 1 (SOD1; #ab13498), Klotho (#ab203576), CD68 (#ab125212) (abcam, Cambridge, MA, USA), NFκB-p65 (#sc-8008), cyclooxygenase (Cox)-2 (#sc1745), β-actin (#sc-47778) (Santa Cruz, Dallas, TX, USA), inducible nitric oxide synthase (iNOS; #610329) (BD, Franklin Lakes, NJ, USA), and Ly6G (#14-5931-82) (eBioscience, San Diego, CA), and then incubated with horseradish peroxidase-conjugated secondary antibodies (Bio-Rad, Hercules, CA, USA). The signals were detected by using enhanced chemiluminescence substrates (Bio-Rad), and developed with a Fuji Blue X-Ray Film. Protein bands were quantitated by using ImageJ software. The raw data/full blot summary is available in the Supplementary Fig. 5.

### Malondialdehyde (MDA) assay

A TBARS assay kit (Cayman, Ann Arbor, MI, USA) was used to determine the oxidative stress in the kidney induced by IRI. The absorbance was read by a spectrophotometer at 530 nm.

### Real-time reverse transcription-polymerase chain reaction (RT-PCR)

Five μg of total RNA were added to a 30 μL reaction volume of the Promega reverse transcriptase reagent mixture. The RT products (100 ng) were as a template for amplification using a SYBR Green PCR amplification reagent (Qiagen). The primer sets for CHOP (forward: 5′-ATGCCCATCTTCTGCTTGTCA-3′, reverse: 5′-CCTTGTAGTTGTGGGTCTTGT-3′), IL-6 (forward: 5′-GCTACCAAACTGGATATAATCAGGA-3′, reverse: 5′-CCAGGTAGCTATGGTACTCCAGAA-3′), β-actin (forward: 5′-CCTGTATGCCTCTGGCGTA-3′, reverse: 5′-CCATCTCTTGCTCGAAGTCT-3′), and Gapdh (forward: 5′-AAGAGGGATGCTGCCCTTAC-3′, reverse: 5′-CCATTTTGTCTACGGGACGA-3′ were used. The mRNA expression was determined by Bio-Rad iQ5 Real-time RT-PCR Detection System (Hercules, CA, USA). The mRNA expression was normalized by the β-actin and Gapdh.

### Immunohistochemistry

The renal tissue sections were deparaffinized and rehydrated. Tissue slides were blocked with bovine serum albumin (1%) for 1 h and then immersed in PBS containing antibodies for CHOP (#2895), cleaved caspase-3 (#9664) (Cell Signalling), Cox-2 (#sc1745) (Santa Cruz), catalase (#ab16731) (abcam), and Ly6G (#14-5931-82) (eBioscience) overnight. Tissue slides were immersed in PBS containing biotin-conjugated secondary antibody for 1 h, and then incubated in streptavidin–horseradish peroxidase solution. The 3,3′diaminobenzidine was used to react with horseradish peroxidase, and brown deposits were formed.

### Statistics

The results are expressed as the mean ± SEM. The significant difference was assessed by one-way analysis of variance with a Dunnett's post hoc test. For the cell culture experiments, a two-way ANOVA followed by post hoc analysis with the Sidak test was used. The *p* values < 0.05 was considered statistically significant.

## Supplementary information

Supplementary file1

## References

[1] Piper RJ, Hughes MA, Moran CM, Kandasamy J (2016). Focused ultrasound as a non-invasive intervention for neurological disease: a review. Br. J. Neurosurg..

[2] Xin Z (2016). Clinical applications of low-intensity pulsed ultrasound and its potential role in urology. Transl. Androl. Urol..

[3] Romano CL, Romano D, Logoluso N (2009). Low-intensity pulsed ultrasound for the treatment of bone delayed union or nonunion: a review. Ultrasound Med. Biol..

[4] Katiyar A, Duncan RL, Sarkar K (2014). Ultrasound stimulation increases proliferation of MC3T3-E1 preosteoblast-like cells. J. Ther. Ultrasound.

[5] Chan CW (2006). Low intensity pulsed ultrasound accelerated bone remodeling during consolidation stage of distraction osteogenesis. J. Orthop. Res..

[6] Cook SD (2001). Improved cartilage repair after treatment with low-intensity pulsed ultrasound. Clin. Orthop. Relat. Res..

[7] Chen CM (2018). Preventive effect of low intensity pulsed ultrasound against experimental cerebral ischemia/reperfusion injury via apoptosis reduction and brain-derived neurotrophic factor induction. Sci. Rep..

[8] Ogata T (2017). Low-intensity pulsed ultrasound enhances angiogenesis and ameliorates contractile dysfunction of pressure-overloaded heart in mice. PLoS ONE.

[9] Gigliotti JC (2013). Ultrasound prevents renal ischemia-reperfusion injury by stimulating the splenic cholinergic anti-inflammatory pathway. J. Am. Soc. Nephrol..

[10] Hull TD, Agarwal A, Hoyt K (2017). New ultrasound techniques promise further advances in AKI and CKD. J. Am. Soc. Nephrol..

[11] Malek M, Nematbakhsh M (2015). Renal ischemia/reperfusion injury; from pathophysiology to treatment. J. Renal Inj. Prev..

[12] Thadhani R, Pascual M, Bonventre JV (1996). Acute renal failure. N. Engl. J. Med..

[13] van den Akker EK (2013). Protection against renal ischemia-reperfusion injury by ischemic postconditioning. Transplantation.

[14] Salahudeen AK, Clark EC, Nath KA (1991). Hydrogen peroxide-induced renal injury: a protective role for pyruvate in vitro and in vivo. J. Clin. Invest..

[15] Chen BL (2015). CCAAT-enhancer-binding protein homologous protein deficiency attenuates oxidative stress and renal ischemia-reperfusion injury. Antioxid. Redox. Signal.

[16] Singh I, Gulati S, Orak JK, Singh AK (1993). Expression of antioxidant enzymes in rat kidney during ischemia-reperfusion injury. Mol. Cell Biochem..

[17] Miller DL (2012). Overview of therapeutic ultrasound applications and safety considerations. J. Ultrasound Med..

[18] Tsuruta JK (2012). Therapeutic ultrasound as a potential male contraceptive: power, frequency and temperature required to deplete rat testes of meiotic cells and epididymides of sperm determined using a commercially available system. Reprod. Biol. Endocrinol..

[19] Quoilin C (2014). Evidence of oxidative stress and mitochondrial respiratory chain dysfunction in an in vitro model of sepsis-induced kidney injury. Biochim. Biophys. Acta..

[20] Granger DN, Kvietys PR (2015). Reperfusion injury and reactive oxygen species: the evolution of a concept. Redox. Biol..

[21] Paller MS, Hoidal JR, Ferris TF (1984). Oxygen free radicals in ischemic acute renal failure in the rat. J. Clin. Invest..

[22] Ruiz S, Pergola PE, Zager RA, Vaziri ND (2013). Targeting the transcription factor Nrf2 to ameliorate oxidative stress and inflammation in chronic kidney disease. Kidney Int..

[23] Nath KA, Norby SM (2000). Reactive oxygen species and acute renal failure. Am. J. Med..

[24] Chen CF, Tsai SY, Ma MC, Wu MS (2003). Hypoxic preconditioning enhances renal superoxide dismutase levels in rats. J. Physiol..

[25] Malhotra JD, Kaufman RJ (2011). ER stress and its functional link to mitochondria: role in cell survival and death. Cold Spring Harb. Perspect. Biol..

[26] Korsmeyer SJ (1993). Bcl-2/Bax: a rheostat that regulates an anti-oxidant pathway and cell death. Semin. Cancer Biol..

[27] Yu W (2013). Berberine protects human renal proximal tubular cells from hypoxia/reoxygenation injury via inhibiting endoplasmic reticulum and mitochondrial stress pathways. J. Transl. Med..

[28] Xu Y (2016). Endoplasmic reticulum stress and its effects on renal tubular cells apoptosis in ischemic acute kidney injury. Ren. Fail..

[29] Zeeshan HM, Lee GH, Kim HR, Chae HJ (2016). Endoplasmic reticulum stress and associated ROS. Int. J. Mol. Sci..

[30] Bandow K (2007). Low-intensity pulsed ultrasound (LIPUS) induces RANKL, MCP-1, and MIP-1beta expression in osteoblasts through the angiotensin II type 1 receptor. J. Cell Physiol..

[31] Lee HT, Ota-Setlik A, Fu Y, Nasr SH, Emala CW (2004). Differential protective effects of volatile anesthetics against renal ischemia-reperfusion injury in vivo. Anesthesiology.

[32] Motayagheni N, Phan S, Eshraghi C, Nozari A, Atala A (2017). A review of anesthetic effects on renal function: potential organ protection. Am. J. Nephrol..

[33] Gigliotti JC, Okusa MD (2014). The spleen: the forgotten organ in acute kidney injury of critical illness. Nephron. Clin. Pract..

[34] Gigliotti JC (2015). Ultrasound modulates the splenic neuroimmune axis in attenuating AKI. J. Am. Soc. Nephrol..

[35] Skrypnyk NI, Harris RC, de Caestecker MP (2013). Ischemia-reperfusion model of acute kidney injury and post injury fibrosis in mice. J. Vis. Exp..

[36] Hayes BT, Merrick MA, Sandrey MA, Cordova ML (2004). Three-MHz ultrasound heats deeper into the tissues than originally theorized. J. Athl. Train..

[37] Chen HA (2019). The antifibrotic and anti-inflammatory effects of icariin on the kidney in a unilateral ureteral obstruction mouse model. Phytomedicine.

