# Supplementary material for: Prevention of acute kidney injury by low intensity pulsed ultrasound via anti-inflammation and anti-apoptosis
Source: Sci Rep. 2020 Aug 31;10:14317. doi: 10.1038/s41598-020-71330-1 (PMC7459306; doi:10.1038/s41598-020-71330-1)

Supplementary Information

Prevention of Acute Kidney Injury by Low Intensity Pulsed Ultrasound via anti-inflammation and anti-apoptosis

Chih-Kang Chiang,[1,2,†] Jui-Zhi Loh,[1,†] Ting-Hua Yang,[3,†] Kuo-Tong Huang,[4] Cheng-Tien Wu,[5,6] Siao-Syun Guan,[7] Shing-Hwa Liu,[1,8,9,*] Kuan-Yu Hung[4,*]

Supplementary Figure 1.

Effects of LIPUS on hypoxia/reoxygenation (H/R)-induced cell viability inhibition in renal tubule NRK52E cells. Cells were treated with H/R (6 h/24 h) with or without LIPUS treatment. LIPUS (100 mW/cm^2) was performed to the cell culture for a period of 15 min before the beginning of the experiment (H/R). The cell viability was determined by MTT assay. Data are presented as means ± SEM (n = 7). *p < 0.05 versus control. #p < 0.05 versus H/R alone.

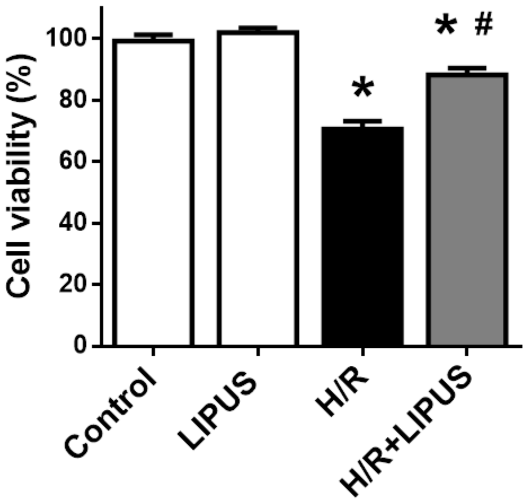

Supplementary Figure 2.

Effects of LIPUS on the renal injury in an acute kidney injury (AKI) mouse model of unilateral ischemia/reperfusion injury (IRI) with contralateral nephrectomy. Animals were euthanized 48 hours after IRI or sham surgery with or without LIPUS treatment. The LIPUS treatment (100 mW/cm^2 intensity) was performed before IRI procedure and after IRI until the day of euthanization. (A) The serum blood urea nitrogen (BUN, a) and creatinine (b) levels were shown. (B) Renal tissues were stained with Periodic Acid-Schiff (PAS) and pathological changes were observed under light microscope. Histological score was also recorded. Data are presented as mean±SEM (n=4). **p* < 0.05 versus sham; # *p* < 0.05 versus IRI alone. Scale bar: 50 μm.

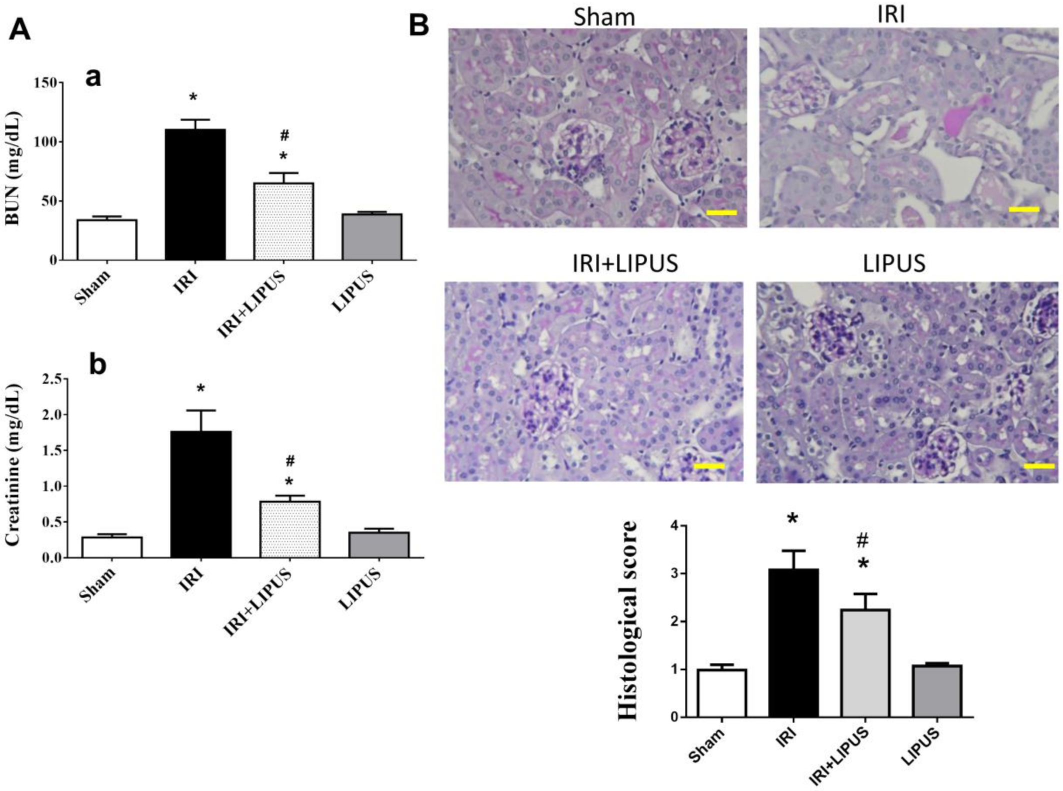

Supplementary Figure 3.

LIPUS by itself did not affect the renal function and morphology in sham control mice. The normal control mice with or without LIPUS treatment were euthanized 7 days after sham surgery. The LIPUS treatment (30 and 100 mW/cm^2 intensity) was performed before sham surgery and after sham surgery until the day of euthanization. (A) The serum blood urea nitrogen (BUN, a) and creatinine (b) levels were shown. (B) Renal tissues were stained with Periodic Acid-Schiff (PAS) and pathological changes were observed under light

microscope. Histological score was also recorded. Data are presented as mean±SEM (n=4). Scale bar: 100 μm.

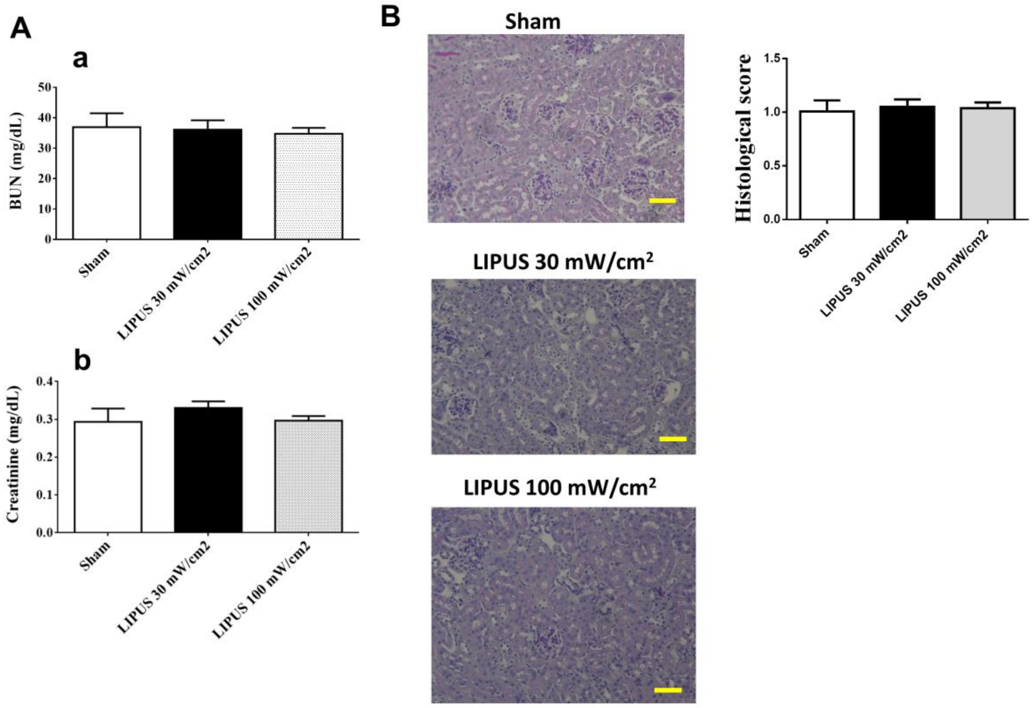

Supplementary Figure 4.

Schematic representation of induction of unilateral IRI mice with contralateral nephrectomy in the presence or absence of LIPUS treatment.

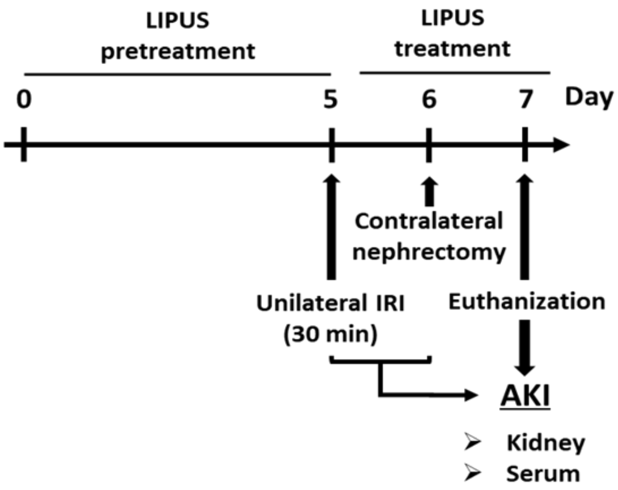

Supplementary Figure 5.
Western blot raw data

(1). Cell model: C: control, H: H2O2 or hypoxia/reoxygenation (H/R), L: LIPUS

(2). AKI (IRI, 48 h) mouse model: S: sham, IRI: IRI alone, L: IRI+LIPUS

(1). Cell model

1. Figure 1B

Treatment: H2O2 for 24 h (Control, H2O2, H2O2+LIPUS, LIPUS); LIPUS 100 mW/cm^2
Tissue: NRK52E cells
Protein: iNOS (130 kDa)

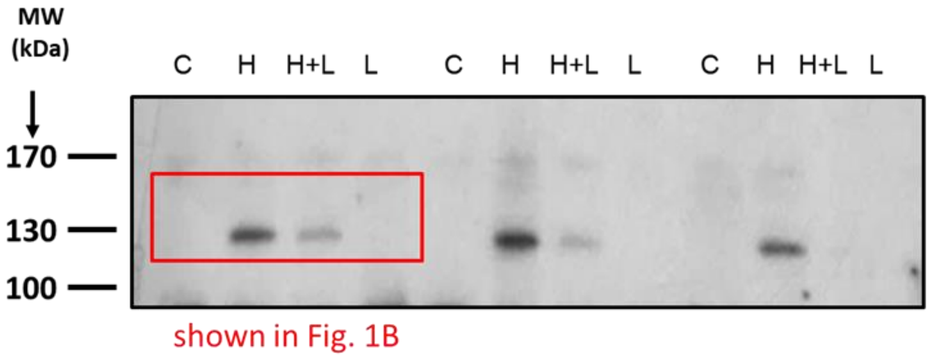

2. Figure 1B

Treatment: H2O2 for 24 h (Control, H2O2, H2O2+LIPUS, LIPUS); LIPUS 100 mW/cm^2
Tissue: NRK52E cells
Protein: Cox-2 (72 kDa)

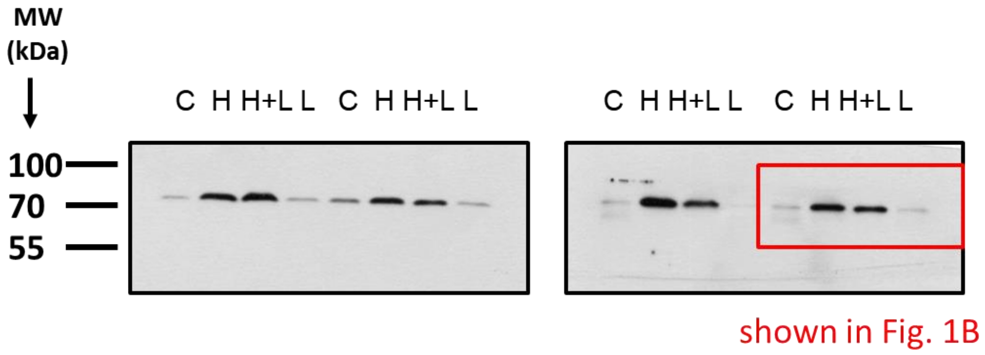

3. Figure 1B

Treatment: H2O2 for 24 h (Control, H2O2, H2O2+LIPUS, LIPUS); LIPUS 100 mW/cm^2
Tissue: NRK52E cells
Protein: p-p65 (65 kDa)

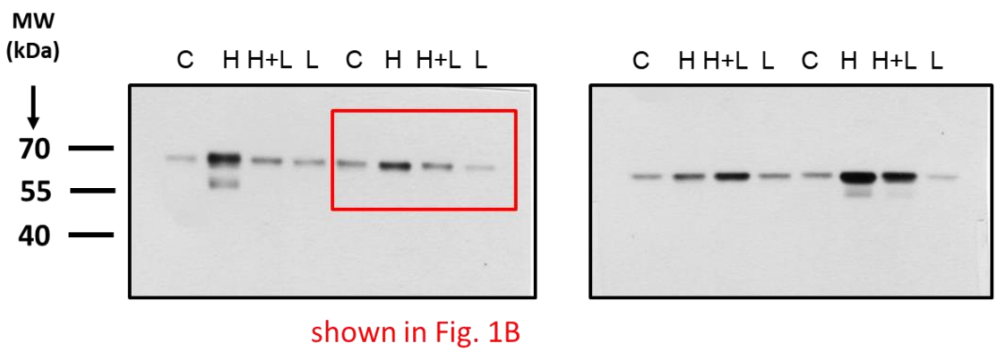

4. Figure 1B

Treatment: H2O2 for 24 h (Control, H2O2, H2O2+LIPUS, LIPUS); LIPUS 100 mW/cm²
Tissue: NRK52E cells
Protein: p65 (65 kDa)

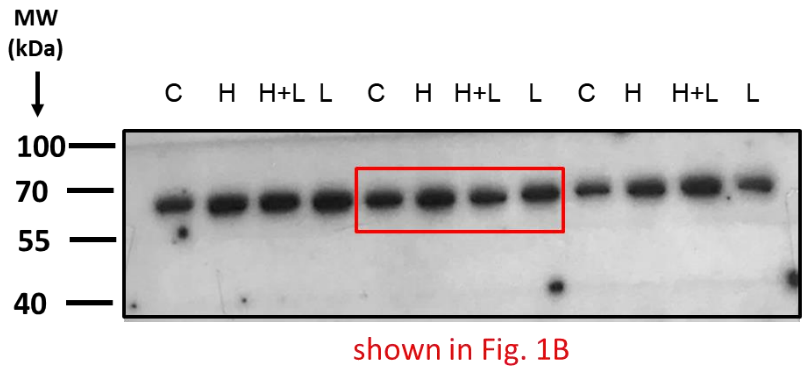

5. Figure 1B

Treatment: H2O2 for 24 h (Control, H2O2, H2O2+LIPUS, LIPUS); LIPUS 100 mW/cm²
Tissue: NRK52E cells
Protein: β-actin (43 kDa)

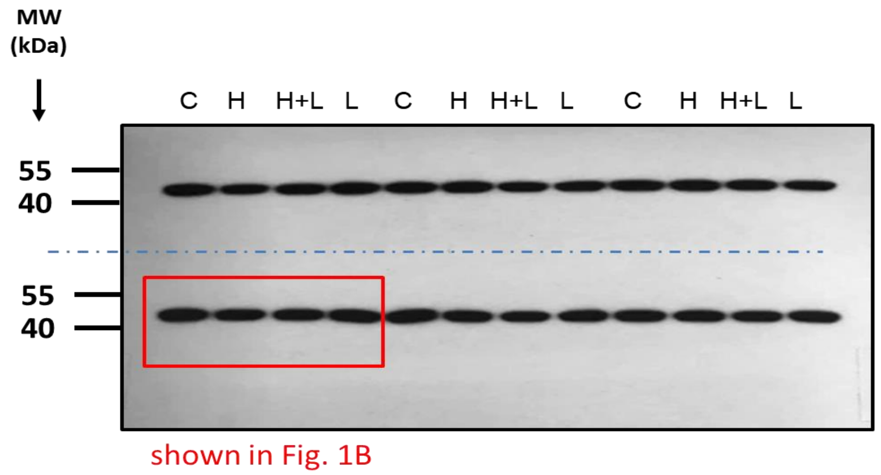

6. Figure 2A

Treatment: H/R (6h/24h) [Control, H/R (H), H/R+LIPUS, LIPUS]; LIPUS 30 mW/cm²
Tissue: NRK52E cells
Protein: Bax (20 kDa)

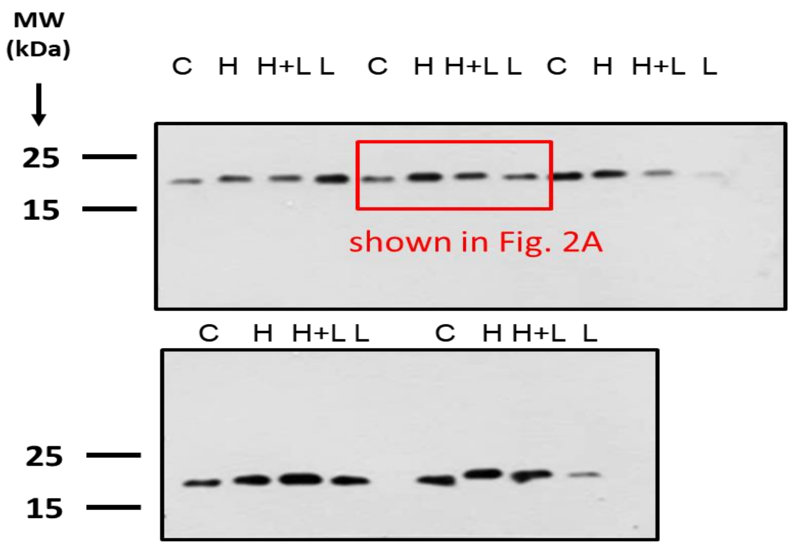

7. Figure 2A

Treatment: H/R (6h/24h) [Control, H/R (H), H/R+LIPUS, LIPUS]; LIPUS 30 mW/cm²
Tissue: NRK52E cells
Protein: Bcl-2 (26 kDa)

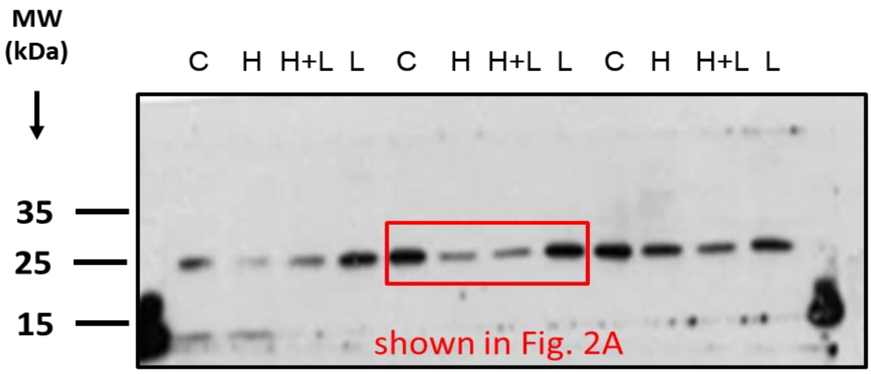

8. Figure 2A

Treatment: H/R (6h/24h) [Control, H/R (H), H/R+LIPUS, LIPUS]; LIPUS 30 mW/cm²
Tissue: NRK52E cells
Protein: CHOP (27 kDa)

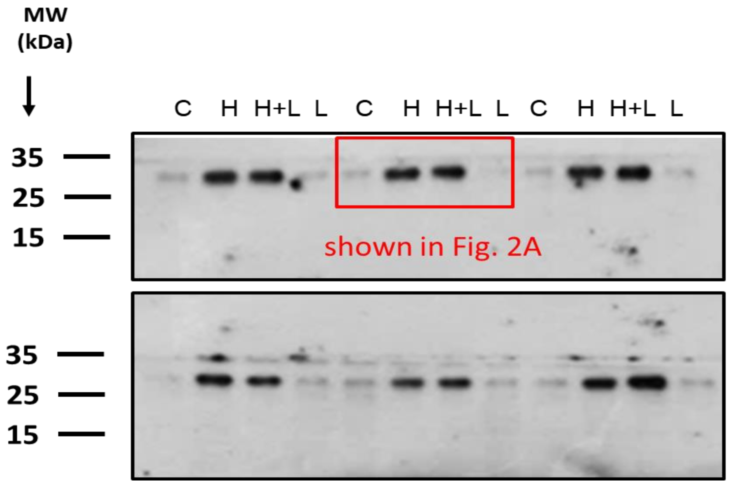

9. Figure 2A

Treatment: H/R (6h/24h) [Control, H/R (H), H/R+LIPUS, LIPUS]; LIPUS 30 mW/cm²
Tissue: NRK52E cells
Protein: cleaved caspase-3 (17 kDa)

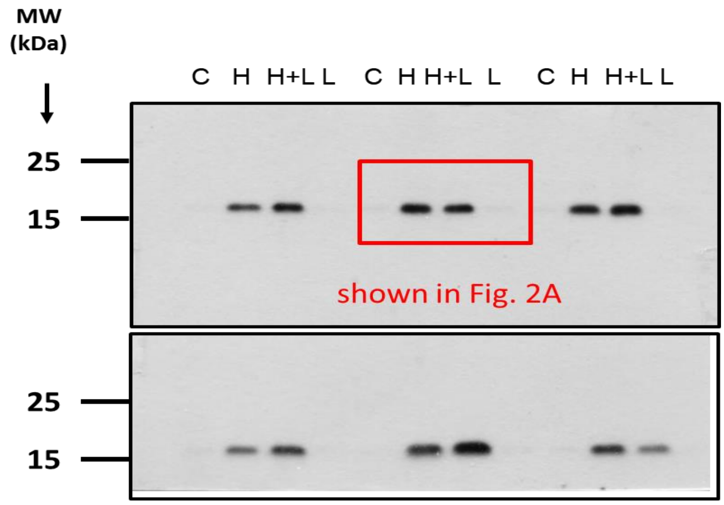

10. Figure 2A

Treatment: H/R (6h/24h) [Control, H/R (H), H/R+LIPUS, LIPUS]; LIPUS 30 mW/cm^2
Tissue: NRK52E cells
Protein: COX-2 (72 kDa)

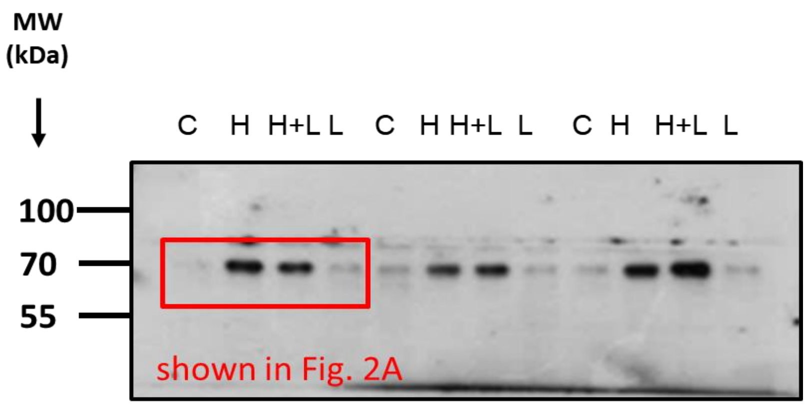

11. Figure 2A

Treatment: H/R (6h/24h) [Control, H/R (H), H/R+LIPUS, LIPUS]; LIPUS 100 mW/cm^2
Tissue: NRK52E cells
Protein: β-actin (43 kDa)

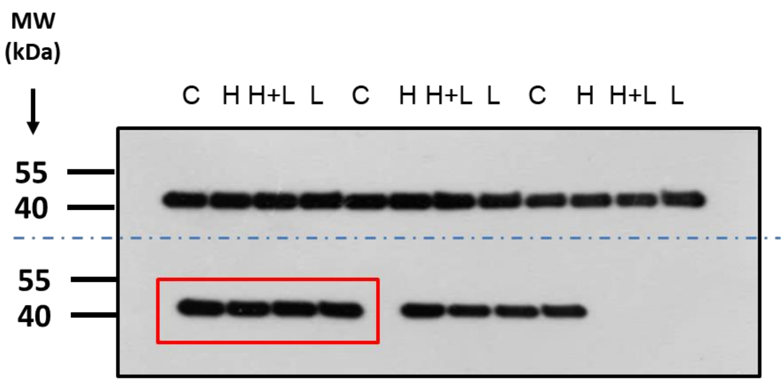

shown in Fig. 2A

12. Figure 2B

Treatment: H/R (6h/24h) [Control, H/R (H), H/R+LIPUS, LIPUS]; LIPUS 100 mW/cm^2
Tissue: NRK52E cells
Protein: Bax (20 kDa)

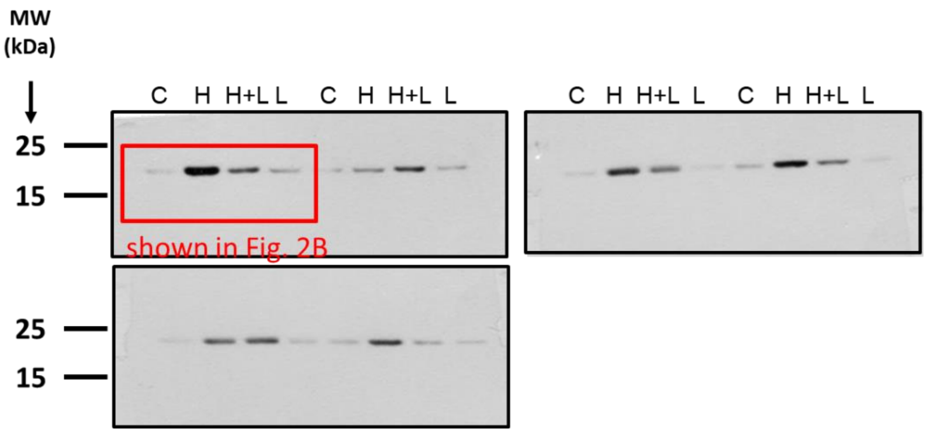

13. Figure 2B

Treatment: H/R (6h/24h) [Control, H/R (H), H/R+LIPUS, LIPUS]; LIPUS 100 mW/cm^2
Tissue: NRK52E cells
Protein: Bcl-2 (26 kDa)

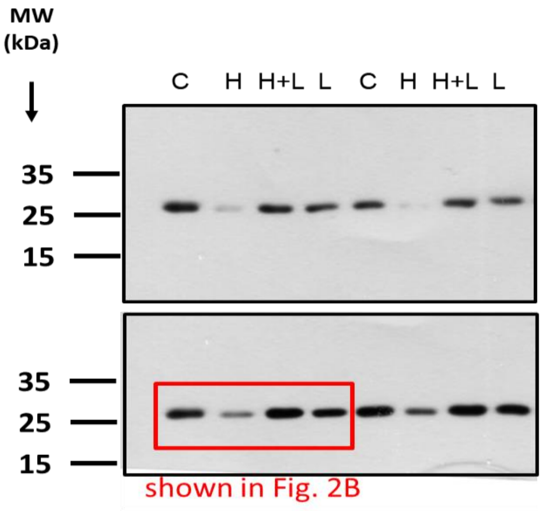

14. Figure 2B

Treatment: H/R (6h/24h) [Control, H/R (H), H/R+LIPUS, LIPUS]; LIPUS 100 mW/cm^2
Tissue: NRK52E cells
Protein: CHOP (27 kDa)

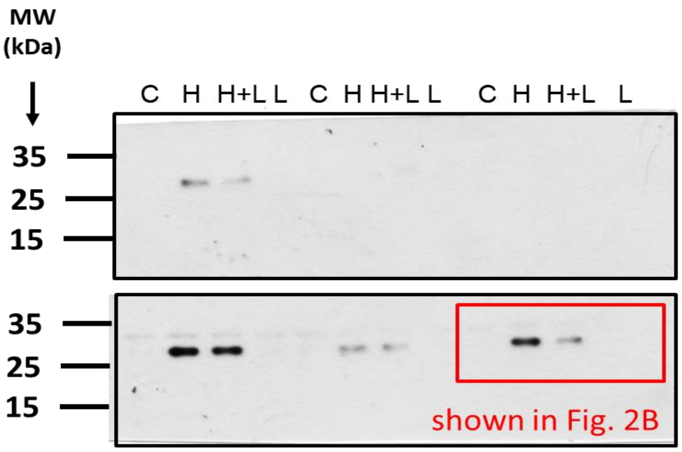

15. Figure 2B

Treatment: H/R (6h/24h) [Control, H/R (H), H/R+LIPUS, LIPUS]; LIPUS 100 mW/cm²
Tissue: NRK52E cells
Protein: cleaved caspase-3 (17 kDa)

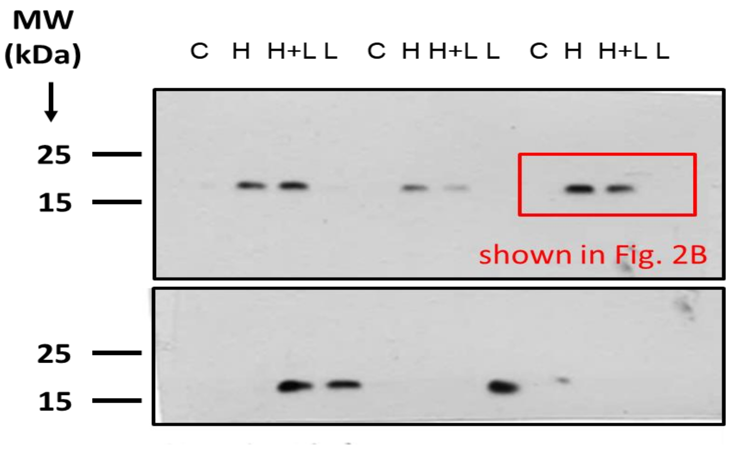

16. Figure 2B

Treatment: H/R (6h/24h) [Control, H/R (H), H/R+LIPUS, LIPUS]; LIPUS 100 mW/cm²
Tissue: NRK52E cells
Protein: COX-2 (72 kDa)

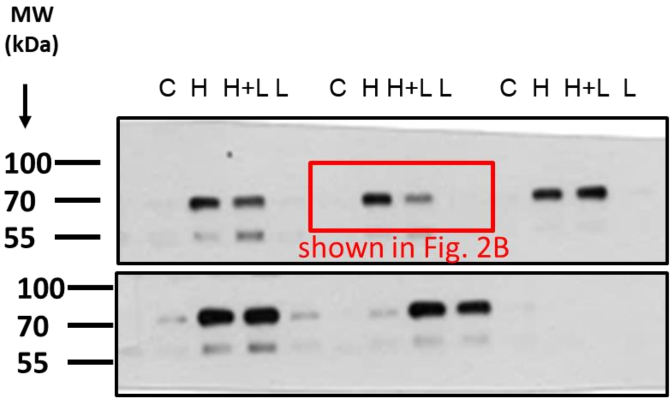

17. Figure 2B

Treatment: H/R (6h/24h) [Control, H/R (H), H/R+LIPUS, LIPUS]; LIPUS 100 mW/cm²
Tissue: NRK52E cells
Protein: β-actin (43 kDa)

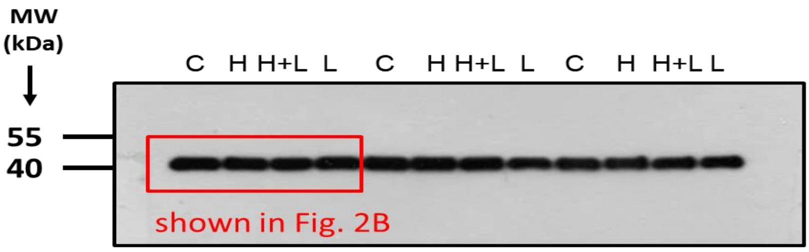

18. Figure 3

Treatment: H/R (6h/24h) [Control, H/R (H), H/R+LIPUS, LIPUS]; LIPUS 100 mW/cm²
Tissue: NRK52E cells
Protein: SOD1 (18 kDa)

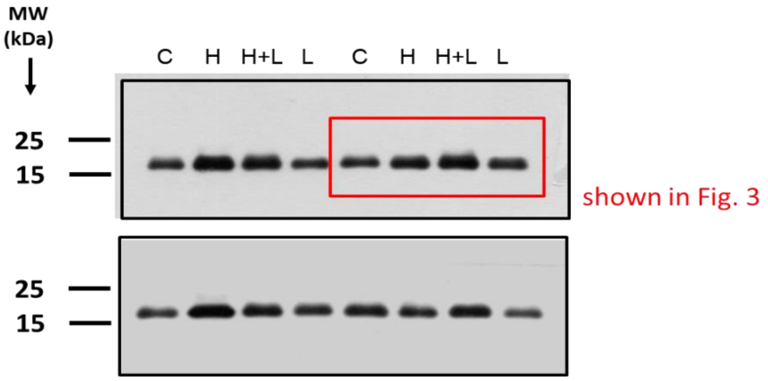

19. Figure 3

Treatment: H/R (6h/24h) [Control, H/R (H), H/R+LIPUS, LIPUS]; LIPUS 100 mW/cm²
Tissue: NRK52E cells
Protein: catalase (60 kDa)

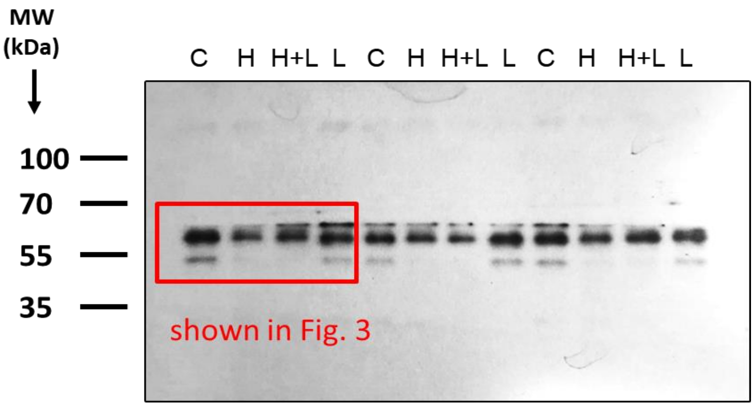

20. Figure 3

Treatment: H/R (6h/24h) [Control, H/R (H), H/R+LIPUS, LIPUS]; LIPUS 100 mW/cm²
Tissue: NRK52E cells
Protein: α-Klotho (116 kDa)

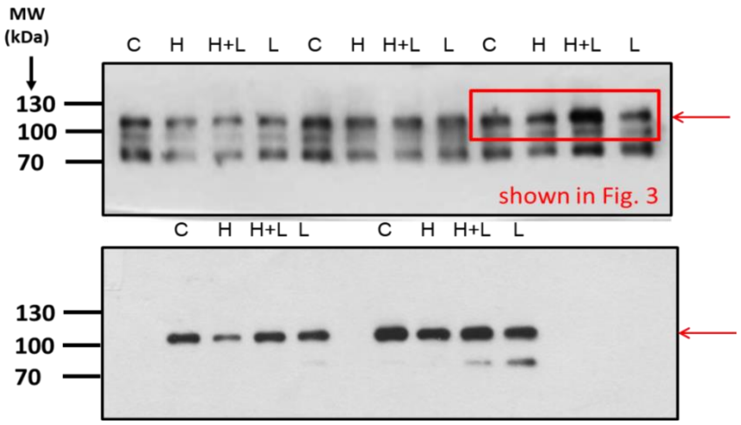

21. Figure 3

Treatment: H/R (6h/24h) [Control, H/R (H), H/R+LIPUS, LIPUS]; LIPUS 100 mW/cm²
Tissue: NRK52E cells
Protein: Sirt1 (120 kDa)

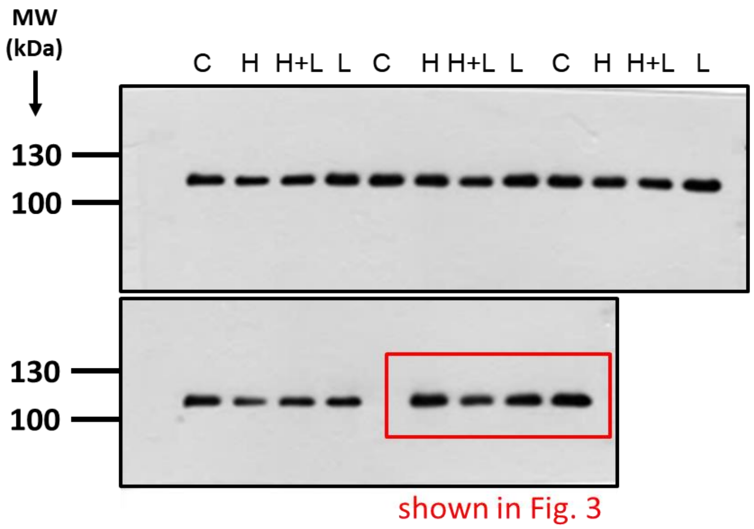

22. Figure 3

Treatment: H/R (6h/24h) [Control, H/R (H), H/R+LIPUS, LIPUS]; LIPUS 100 mW/cm²
Tissue: NRK52E cells
Protein: β-actin (43 kDa)

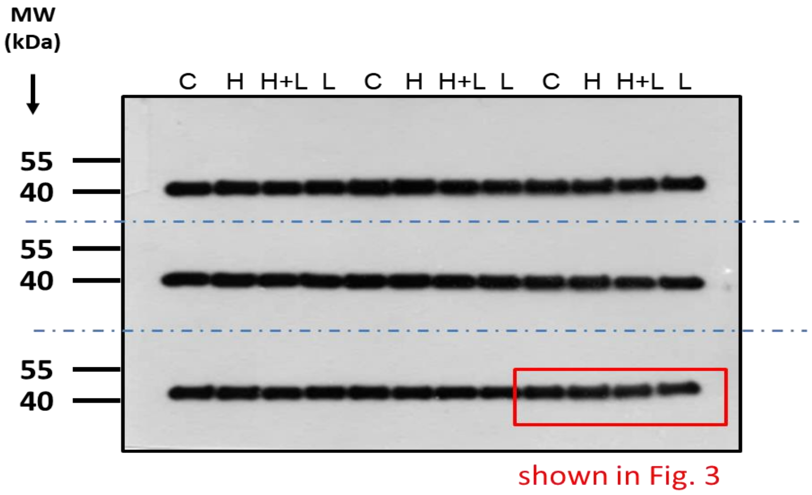

(2). AKI (IRI 48 h) mouse model

1. Figure 5

Treatment: AKI-48h (sham, IRI, IRI+LIPUS)
Tissue: Kidney
Protein: GRP78 (78 kDa)

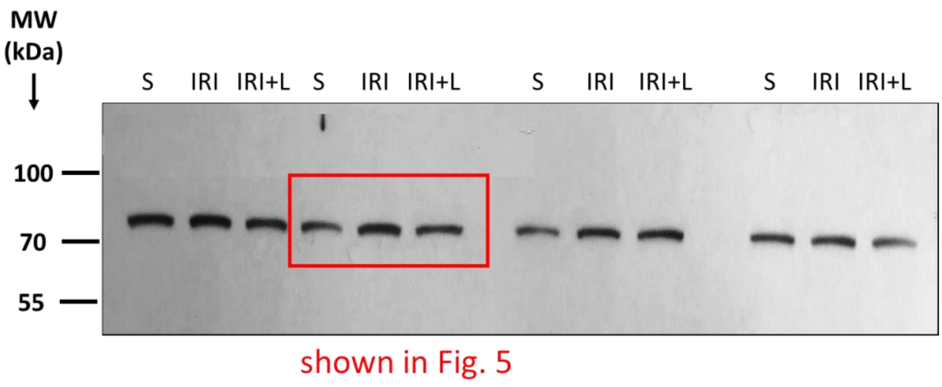

2. Figure 5

Treatment: AKI-48h (sham, IRI, IRI+LIPUS)
Tissue: Kidney
Protein: CHOP (27 kDa)

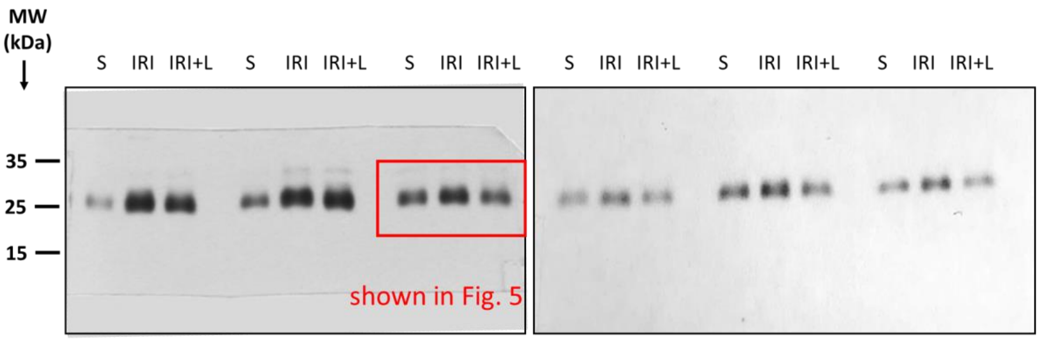

3. Figure 5

Treatment: AKI-48h (sham, IRI, IRI+LIPUS)
Tissue: Kidney
Protein: Bcl-2 (26 kDa)

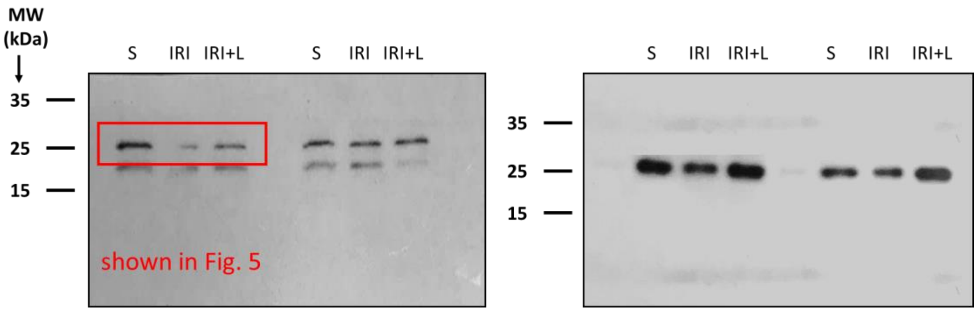

4. Figure 5

Treatment: AKI-48h (sham, IRI, IRI+LIPUS)
Tissue: Kidney
Protein: Bax (20 kDa)

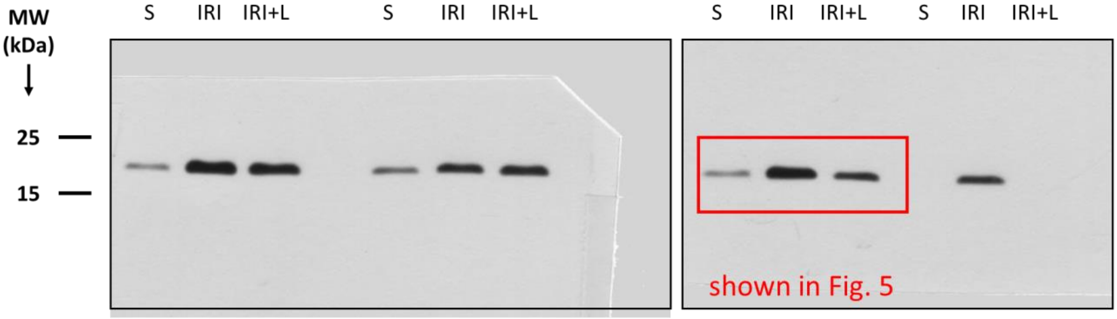

5. Figure 5

Treatment: AKI-48h (sham.IRI.IRI+LIPUS)
Tissue: Kidney
Protein: Cleaved Caspase-3 (17, 19 kDa)

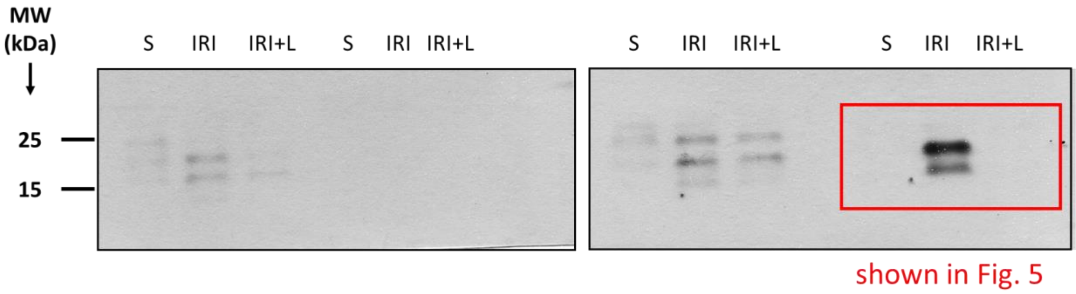

6. Figure 5

Treatment: AKI-48h (sham.IRI.IRI+LIPUS)
Tissue: Kidney
Protein: COX2 (72 kDa)

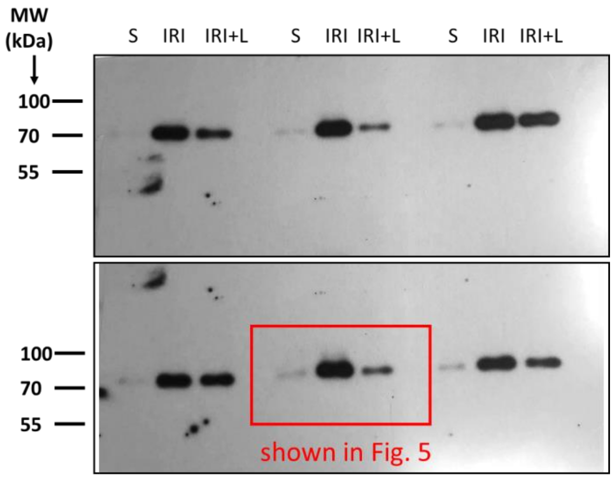

7. Figure 6A

Treatment: AKI-48h (sham, IRI, IRI+LIPUS)
Tissue: Kidney
Protein: SOD1 (18 kDa)

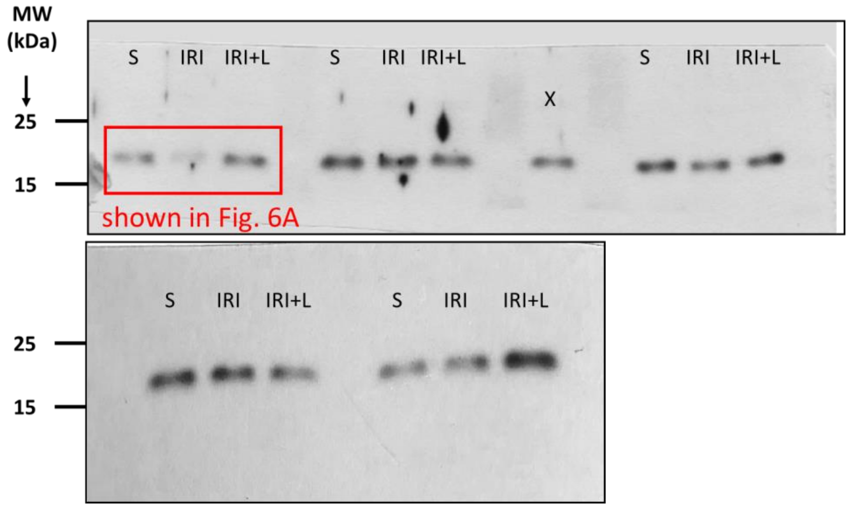

8. Figure 6A

Treatment: AKI-48h (sham, IRI, IRI+LIPUS)
Tissue: Kidney
Protein: catalase (60 kDa)

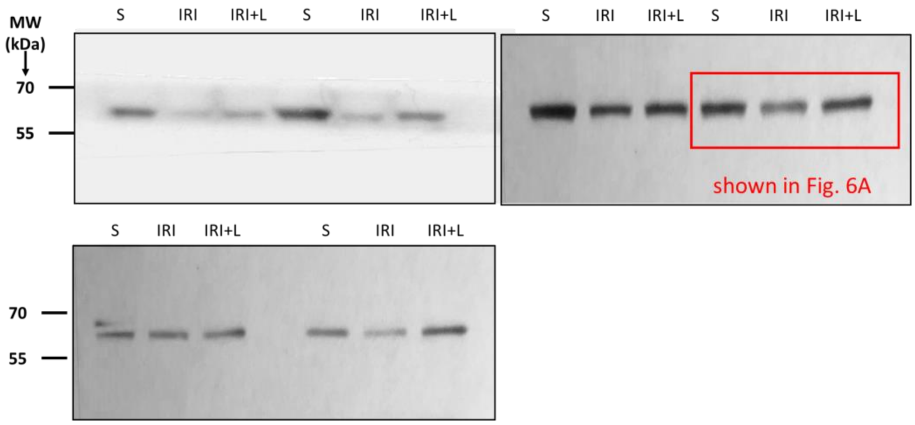

9. Figures 5 and 6A

Treatment: AKI-48h (sham, IRI, IRI+LIPUS)
Tissue: Kidney
Protein: β-actin (43 kDa)

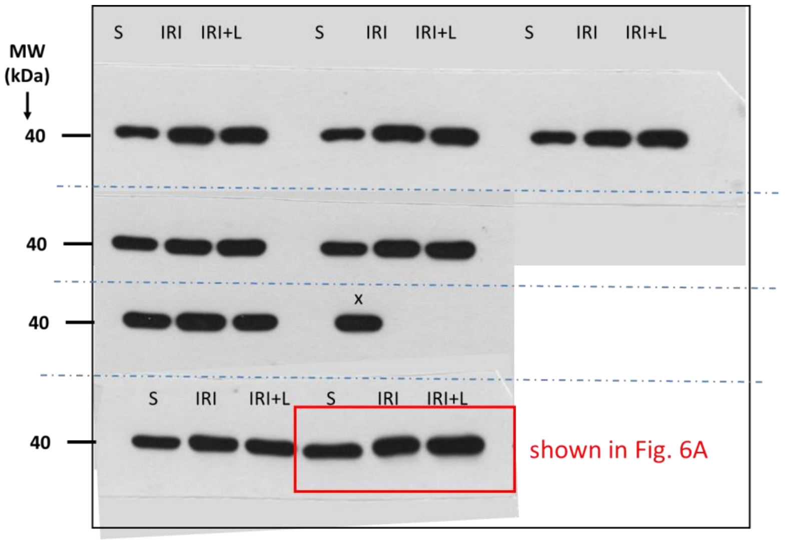

10. Figure 8A-a

Treatment: AKI-48h (sham, IRI, IRI+LIPUS)
Tissue: Kidney
Protein: Ly6g (21-25 kDa)

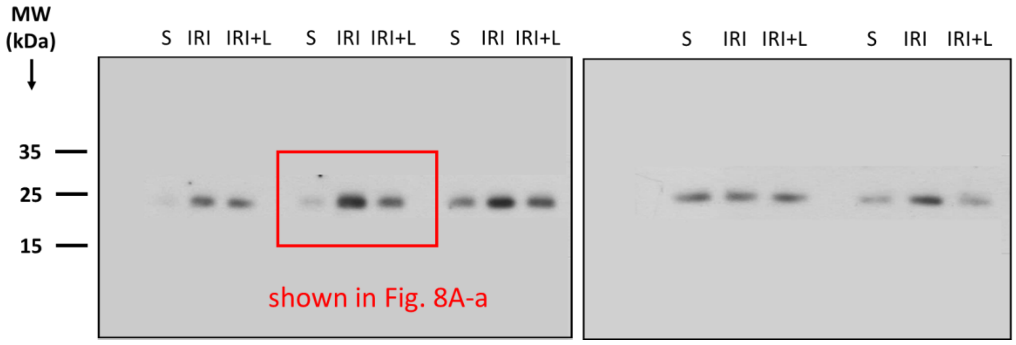

11. Figure 8A-a

Treatment: AKI-48h (sham, IRI, IRI+LIPUS)
Tissue: Kidney
Protein: CD68 (polyclonal antibody, predicted molecular weight: 35 kDa)

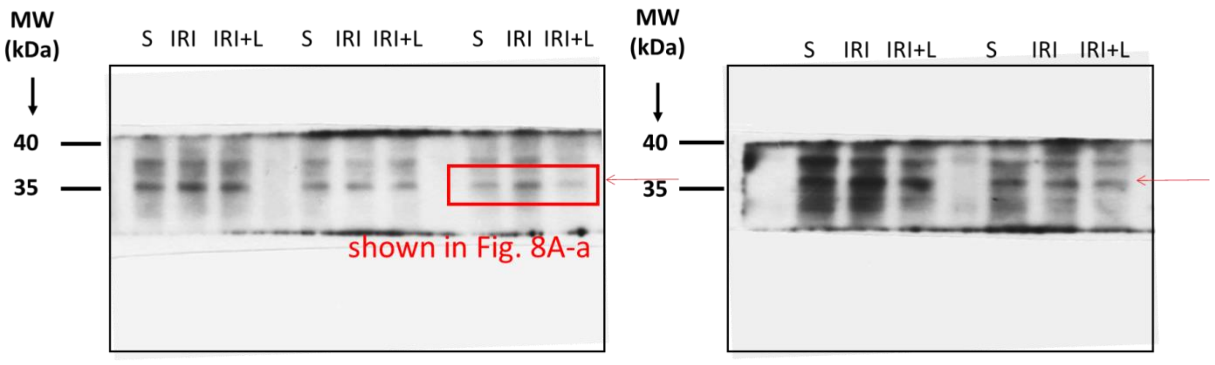

12. Figure 8A-a

Treatment: AKI-48h (sham, IRI, IRI+LIPUS)
Tissue: Kidney
Protein: GAPDH (37 kDa)

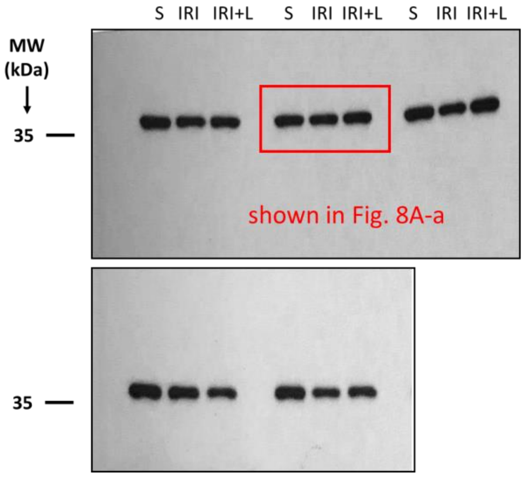

13. Figure 8A-b

Treatment: AKI-48h (sham, IRI, IRI+LIPUS)
Tissue: Spleen
Protein: Ly6g (21-25 kDa)

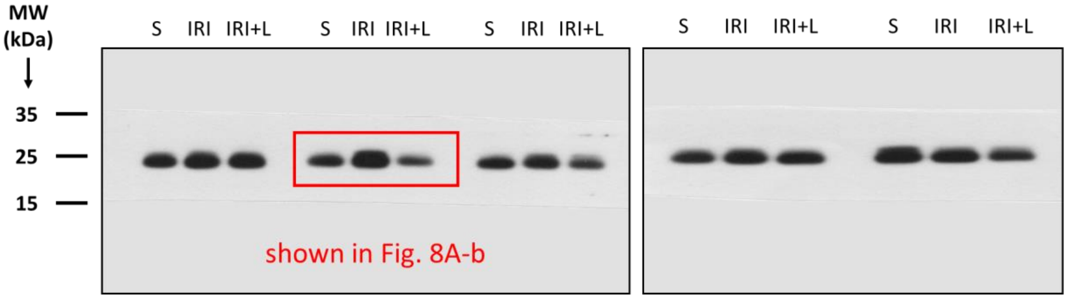

14. Figure 8A-b

Treatment: AKI-48h (sham, IRI, IRI+LIPUS)
Tissue: Spleen
Protein: CD68 (polyclonal antibody, predicted molecular weight: 35 kDa)

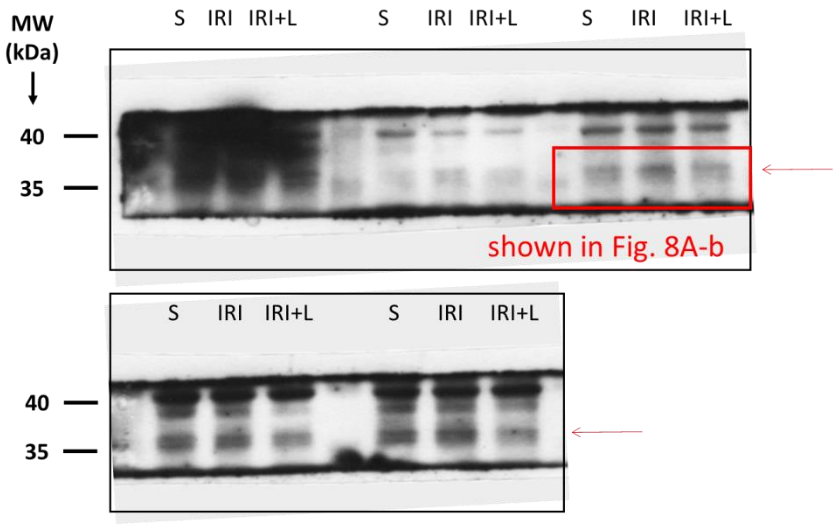

15. Figure 8A-b

Treatment: AKI-48h (sham, IRI, IRI+LIPUS)
Tissue: Spleen
Protein: GAPDH (37 kDa)

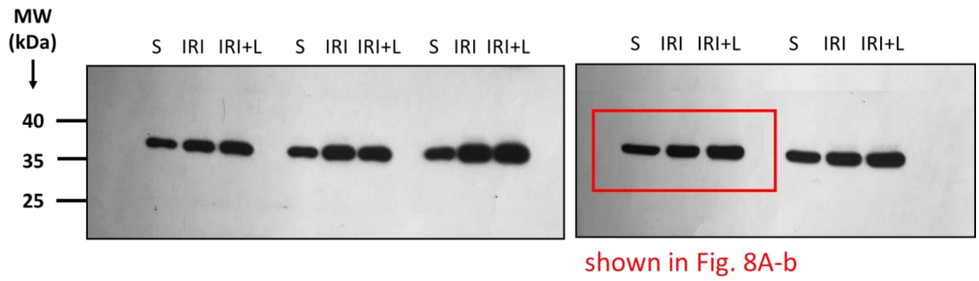

Supplement: Supplementary file 1 — Supplementary file1 [file 41598_2020_71330_MOESM1_ESM.pdf]
